# Revealing the metabolic potential and environmental adaptation of nematophagous fungus, *Purpureocillium lilacinum*, derived from hadal sediment

**DOI:** 10.3389/fmicb.2024.1474180

**Published:** 2024-11-06

**Authors:** Yongqi Li, Changhao Zhang, Maosheng Zhong, Shenao Hu, Yukun Cui, Jiasong Fang, Xi Yu

**Affiliations:** ^1^Shanghai Engineering Research Center of Hadal Science and Technology, College of Oceanography and Ecological Science, Shanghai Ocean University, Shanghai, China; ^2^Laboratory for Marine Biology and Biotechnology, Qingdao Marine Science and Technology Center, Qingdao, China

**Keywords:** piezotolerance, hadal fungi, Mariana Trench, adaptation mechanism, transcriptome

## Abstract

The extreme environment shapes fungi in deep-sea sediments with novel metabolic capabilities. The ubiquity of fungi in deep-sea habitats supports their significant roles in these ecosystems. However, there is limited research on the metabolic activities and adaptive mechanisms of filamentous fungi in deep-sea ecosystems. In this study, we investigated the biological activities, including antibacterial, antitumor and nematicidal activity of *Purpureocillium lilacinum* FDZ8Y1, isolated from sediments of the Mariana Trench. A key feature of *P. lilacinum* FDZ8Y1 was its tolerance to high hydrostatic pressure (HHP), up to 110 MPa. We showed that HHP affected its vegetative growth, development, and production of secondary metabolites, indicating the potential for discovering novel natural products from hadal fungi. Whole-genome sequencing of *P. lilacinum* FDZ8Y1 revealed the metabolic potential of this piezotolerant fungus in carbon (carbohydrate metabolism), nitrogen (assimilatory nitrate reduction and protein degradation) and sulfur cycling processes (assimilatory sulfate reduction). Transcriptomic analysis under elevated HHP showed that *P. lilacinum* FDZ8Y1 may activate several metabolic pathways and stress proteins to cope with HHP, including fatty acid metabolism, the antioxidant defense system, the biosynthetic pathway for secondary metabolites, extracellular enzymes and membrane transporters. This study provides valuable insights into the metabolic potential and adaptation mechanisms of hadal fungi to the challenging conditions of the hadal environment.

## Introduction

The hadal zones, regions of the deep sea below 6,000 meters, represent the least-explored aquatic biosphere on Earth, occupying 45% of the vertical depth of the ocean (Jamieson et al., 2010; Jamieson, 2015). Characterized by high hydrostatic pressure (HHP) and isolation from the upper ocean (Tamburini et al., 2013; Nunoura et al., 2015), these environments host a variety of microorganisms, including fungi (Danovaro et al., 2003; Jamieson et al., 2010; Lidbury et al., 2015; Schmidt and Martínez Arbizu, 2015; Zhang et al., 2024). Historically, research has focused on deep-sea prokaryotes (DeLong and Pace, 2001; Sogin et al., 2006), but recent studies have increasingly turned to hadal fungi due to their potential for novel bioactive compounds and ecological roles. Experimental studies have revealed the indispensable role of hadal fungi in their environment, characterized by the unique adaptive mechanisms and metabolic activities they have developed to survive in the challenging deep-sea environments (Raghukumar et al., 2010; Nagahama and Nagano, 2012; Richards et al., 2012; Zhong et al., 2024).

Hadal fungi, with their genetic diversity and adaptation to extreme environments, are a promising source of unique and medicinally important secondary metabolites (SMs) (Wang et al., 2015; Arifeen et al., 2019). A wide range of bioactive molecules have been isolated from deep-sea fungal communities, showing potential for various activities such as antibacterial, antiviral, antidiabetic, anti-inflammatory, and antitumor effects, as well as serving as important enzymes (Shang et al., 2012; Tiquia and Mormile, 2010; Wang et al., 2015; Wang et al., 2020). Recent findings also highlight the profound influence of HHP on the production and bioactivity of SMs by hadal fungi. HHP significantly impacts the production and bioactivity of fungal SMs, potentially enhancing the expression of biosynthetic gene clusters through the activation of environmental stress responses (Deng et al., 2023; Peng et al., 2023). The modulation of the fungal metabolic processes under HHP conditions may result in the generation of novel bioactive substances with unique mechanisms of action, which are vital for fungal survival in the deep sea and also offer promising prospects for the development of new drugs and biotechnological applications (Arifeen et al., 2020). Despite these insights, a more thorough understanding of how hadal fungi adapt metabolically to extreme conditions is essential for fully harnessing their potential in bioactive compound discovery.

Fungi are key intermediates in marine ecosystems, facilitating energy flow from detritus to higher trophic levels (Hyde and Lee, 1995; Xu et al., 2014; Zhang et al., 2021). They dominate bathypelagic marine snow particles, playing a role in carbon and nutrient transport in the deep ocean (Bochdansky et al., 2017; Zvereva and Borzykh, 2022). The adhesive properties of fungal hyphae contribute to humus formation in deep-sea sediments. The abundance, diversity, and expression of fungal peptidases and CAZymes indicate a close coupling between carbohydrate and protein degradation by fungi in the oceans (Morales et al., 2019; Baltar et al., 2021; Breyer et al., 2022). A study on piezotolerant fungi from the Mariana Trench indicates that hadal fungi, which are microorganisms capable of growing at or slightly above atmospheric pressure (0.1–10 MPa) (Fang et al., 2010), possess potential ecological and metabolic functions in hadal trench ecosystems (Li et al., 2022). In deep-sea ecosystems, nematodes dominate the metazoan community, representing over 90% of its total abundance (Danovaro et al., 2008; Danovaro et al., 2014; Snelgrove et al., 2014). There are ecological associations between fungi and nematodes (Bhadury et al., 2011), and limited data from previous studies have suggested these relationships. Recently, a nematophagous fungus, *Purpureocillium lilacinum*, was isolated from the hadal trenches (Li et al., 2022), but further studies are needed to explore its bioactive potential against nematodes.

Here, we selected a piezotolerant fungal isolate, *P. lilacinum*, from the sediments of the Mariana Trench. We evaluated its antibacterial, antitumor, and nematicidal activities through *in vitro* biochemical experiments and genomic predictions. Furthermore, we elucidated the ecological role of this fungus in the deep ocean through genomic and physiological analysis. Transcriptome analysis provided insights into the molecular mechanisms enabling fungi to withstand environmental stress, offering a theoretical foundation for understanding stress resistance in filamentous fungi. This research bridges environmental factors and the ecological functions of hadal microorganisms, providing valuable insights for future studies on applications of hadal fungal resources.

## Materials and methods

### Sediment sampling and isolation of fungus

The sediment sample was collected using a pressure-retaining sampling device from the Challenger Deep, Mariana Trench (142.2148 °E, 11.3403 °N, 10,898 m), located in the Western Pacific Ocean, during the cruise of the Discovery-One research vessel (TS 21) in October 2021. The sediment sample was then aseptically transferred into sterile 50 mL tubes and stored at 4°C until further analysis. To isolate the fungi, the diluted hadal sediment was spread-plated on Emerson YpSs agar medium (EYA) (Supplementary Table S1), and the media were cultivated at 28°C to isolate single fungal colonies. The fungus was inoculated into potato dextrose agar (PDA) (Supplementary Table S1) for further incubation.

### Fungal identification and phylogenetic analysis

The identification of the isolate fungus recovered from the hadal sediment was proposed in the species after combining morphological and molecular data. Genomic DNA was extracted using the TIANcombi DNA Lyse (TIANGEN BIOTECH, China) according to the manufacturer’s instructions. The Internal Transcribed Spacer (ITS) fragment was amplified using a universal primer set for ITS1 / ITS4 (Supplementary Table S2). The polymerase chain reaction (PCR) was conducted as previously described (Deng et al., 2023). The amplified PCR product was sent to GENEWIZ (Suzhou, China) for sequencing and identification against sequences in the NCBI database^1^ using the BLASTn program. The phylogenetic tree was constructed with 1,000 bootstrap replicates in MEGA X (Kumar et al., 2018).

### Evaluation of antibacterial activity and cytotoxic activity of fungal secondary metabolites (SMs)

The fungal colony was cultured on PDA medium at 28°C for 3–7 days. Spore suspensions were prepared and inoculated into potato dextrose broth (PDB) (Supplementary Table S1). Then the fermentation broth was incubated at 28°C for 8 days in a rotary incubator at 180 rpm. The liquid culture of FDZ8Y1 was extracted three times with ethyl acetate of equal volume using ultrasonic-assisted extraction. The combined extract was concentrated using a rotary evaporator to obtain the ethyl acetate crude extract, following the method described by Xiao et al. (2023).

#### Evaluation of antibacterial activity of fungal SMs

The antibacterial activity of the fungal crude extract was evaluated using the Kirby-Bauer disk diffusion method. Indicator bacteria were provided by Shanghai Rainbowfish Company (Supplementary Table S3) and cultured in Luria-Bertani (LB) (Supplementary Table S1) broth. The crude extract was dissolved in the methanol at a final concentration of 100 mg/mL. The antibacterial assay was conducted as previously described (Deng et al., 2023). The methanol was used as the negative control. An inhibition zone was observed after incubation at 37°C for 12 h to evaluate the antibacterial bioactivity of the crude extract of SMs from *P. lilacinum* FDZ8Y1.

#### Evaluation of cytotoxic activity of fungal SMs

The MTT (3-[4,5-dimethylthiazol-2-yl]-2,5-diphenyltetrazolium bromide) assay was used to measure cytotoxicity of the fungal crude extract. Cell lines were obtained from the National Collection of Authenticated Cell Cultures (Shanghai, China) (Supplementary Table S3). All cells were incubated in a 37°C humidified incubator with 5% CO_2_. Cells from the third generation were subsequently employed for further experiment. The crude extract and positive control (hydroxydaunorubicin, Dox) were dissolved in dimethyl sulfoxide (DMSO) to final concentration of 500 μg/mL in 96-well plates. Each well was deposited with 200 μL of cells (3 × 10^3^ per well for cancer cell lines). The negative control was treated with an equal volume of DMSO in each well. All the treatments were replicated in triplicate. After incubating the cell lines at 37°C for 24 h, 20 μL of MTT solution (5 mg/mL, dissolved in 1× PBS) was deposited in each well. 96-well plate was then incubated in a 37°C humidified incubator with 5% CO_2_ for 4 h. Finally, the culture medium was removed, and 100 μL of DMSO was added to each well to dissolve the formed crystals completely. The absorbance was then measured using a microplate reader. The cytotoxic assay was conducted as previously described (Xiao et al., 2023).

### Evaluation of nematicidal potential of hadal fungus

*Caenorhabditis elegans* were cultured on nematode growth medium agar plates (NGM) (Supplementary Table S1) with bacteria *Escherichia coli* OP50. The eggs were collected and dispersed with M9 buffer (Supplementary Table S1) at 20°C. The second-stage *C. elegans* juveniles (J2s) were collected. Fungal SMs were dissolved in methanol to achieve a concentration of 200 mg/mL. Briefly, J2s of *C. elegans* were placed in M9 buffer and exposed to the SMs in micro-well bioassay experiments. Each well was loaded with 100 μL of M9 buffer containing J2s (100) of *C. elegans*, 194 μL of M9 buffer and 6 μL of fungal SMs (200 mg/mL). Wells containing an equal volume of methanol and 1 mg/mL abamectin were used as negative controls and positive controls, respectively. Microwell plates were incubated in the dark at 20°C, and the data on the percent of J2s mortality were recorded after incubating for 24 h.

To investigate the nematicidal ability of fungal mycelium, fungal spores were co-cultured with eggs and juvenile nematodes. The fungal spores were mixed with eggs and juveniles by M9 buffer, respectively, then the mixture was dispersed onto a 35 mm Petri dish containing 2 mL 2% agar. The Petri dishes were incubated in the dark at 20°C and were then observed every 24 h.

Ultra-performance liquid chromatography/tandem mass spectrometry (UPLC-MS/MS) was employed as previously described (Peng et al., 2021) to qualitatively identify the SM of *P. lilacinum* FDZ8Y1. The crude extracts were dissolved in methanol and filtered through 0.22 μm filter membrane to achieve the conditions for UPLC analysis. The crude extracts were characterized by a Vanquish UPLC high-resolution mass spectrometer (Thermo Fisher) using a C18 column (2.1 × 100 mm, 1.7 μm, Waters ACQUITY UPLC BEH). The mobile phase was a gradient elution system comprising water (solvent A) and methanol (solvent B), with a flow rate of 0.3 mL/min. The temperature of the column oven was kept at 45°C. A full scan was run with a mass range from 100 to 1,500 *m/z*. An electrospray ionization (ESI) source was used, and both positive and negative ion modes were applied for the acquisition of primary and secondary mass spectrometry data for compound identification.

### Assay of chitinase activity of hadal fungus

The 100 μL spore suspension (10^7^ spores/mL) was inoculated into a chitinase-producing medium (Supplementary Table S1) and incubated at 28°C in a rotary incubator at 100 rpm. The chitinase activity was quantified by 3, 5-dinitrosalicylic acid (DNS) assays to measure the amount of reducing sugars released from chitin (Breuil and Saddler, 1985). The methods for measuring chitinase activity were performed in accordance with those described by Xie et al. (2021). The reduction in sugar concentration was calculated using the standard curve of glucose, which was then multiplied by the dilution factor. One unit of chitinase activity was defined as the amount of enzyme required to produce 1 μmol reducing sugar in 1 min under the above conditions.

### Piezotolerance of hadal fungal spores and mycelia

High hydrostatic pressure (HHP) represents a crucial parameter within the deep-sea biosphere. To test the stress tolerance of *P. lilacinum* FDZ8Y1 under different hydrostatic pressures, a spore suspension of 4 × 10^6^ spores/mL was prepared, then transferred into a 2 mL sterile syringe. These syringes were suspended in pressure vessel filled with pure water, and pressurized to different hydrostatic pressures (0.1, 20, 40, 60, 80, and 110 MPa) at room temperature, respectively. After 2 weeks of incubation, 2.5 μL of the spore suspension was inoculated onto PDA and incubated at 28°C under atmospheric pressure. The germination rate, viability, the development of mycelial growth and colony morphology was recorded regularly. The piezotolerant assay was conducted as previously described (Deng et al., 2023).

The spores were suspended in PDB for incubation for 3 days at 28°C in a rotary incubator at 180 rpm to develop mycelia. 20 mL of mycelia were transferred into a 20 mL sterile syringe and subjected to different hydrostatic pressures (0.1, 20, 40, 60, 80, and 110 MPa) for 2 weeks. After 2 weeks, 10 fungal mycelium blocks were randomly selected and rinsed three times with sterile water. Subsequently, the selected mycelia were inoculated into PDB and incubated at 28°C under atmospheric pressure to detect mycelial viability. Meanwhile, fungal mycelium blocks were randomly selected and stained with 4′, 6-diamidino-2-phenylindole dihydrochloride (DAPI, Sigma-Aldrich, Shanghai) to determine the nucleus of hyphae under different HHP conditions to evaluate the viability.

### Antibacterial activity assay and high-performance liquid chromatography (HPLC) analysis of fungal SMs under different HHP

The spore suspension was treated under different hydrostatic pressures (0.1, 20, 40, 60, 80, and 110 MPa), respectively. After 2 weeks, 200 μL of the spore suspension was inoculated into PDB and incubated at 28°C for 10 days in a rotary incubator at 180 rpm under atmospheric pressure. The crude extracts were dissolved in methanol to the same concentration of 100 mg/mL to test the antibacterial activity, following the method described above. The dissolved crude extracts were then diluted with methanol and filtered for HPLC analysis. The crude extracts were characterized by Agilent 1,260 HPLC using a C18 column (250 × 9.4 mm, 5 μm, Agilent Zorbax SB). The mobile phase was a gradient elution system comprising water (solvent A) and methanol (solvent B), with a flow rate of 1 mL/min. The column thermostat was kept at 40°C, while the detector was operated at 210 nm.

### Genomic DNA extraction, sequencing, and assembly

Genomic DNA was extracted with the GP1 method (cetyltrimethylammonium bromide, CTAB) according to the manufacturer’s protocol (Healey et al., 2014) to obtain high-quality genomic DNA suitable for downstream sequencing. The integrity and purity of the extracted DNA were assessed by 1% agarose gel electrophoresis. DNA concentration was quantified using a Qubit^®^ 2.0 fluorescence meter (Thermo Scientific). A sequencing library was constructed using the NEBNext^®^ Ultra™ DNA Library Prep Kit for Illumina (NEB, USA) and Single Molecule, Real-Time (SMRT) bell TM Template kit (version 2.0.) following manufacturer’s protocol, respectively. The whole genome of *P. lilacinum* FDZ8Y1 was sequenced using the Illumina NovaSeq PE150 system and analyzed on the PacBio Sequel platform at the Novogene Bioinformatics Technology (Beijing, China). The methods associated with genome component prediction and function annotation were listed in the Supplementary materials.

### Comparative genomics and phylogenomic analysis

A comparative genomic analysis was conducted on *P. lilacinum* FDZ8Y1 and other nematophagous fungi (NF), as listed in Supplementary Table S4. To ascertain the genetic functional differences among the strains, CAZymes, proteases, transporters and secondary metabolite genes were predicted as described above. Orthologous groups from *P. lilacinum* FDZ8Y1 and the other fungi listed in Supplementary Table S4 were identified with OrthoFinder, version 2.5.5^2^ (Emms and Kelly, 2019), utilizing the default parameters. The single-copy orthologues proteins were acquired and aligned with Mafft^3^ (Katoh and Standley, 2013). The alignment sequences were concatenated and subsequently employed for phylogenetic reconstruction via the maximum likelihood method, implemented in the Interactive Tree of Life (ITOL)^4^ (Letunic and Bork, 2021).

### RNA-seq analysis

To perform RNA sequencing and transcriptomics analysis, *P. lilacinum* FDZ8Y1 was incubated at 180 rpm at 28°C on PDB for 3 days. Then mycelia were transferred into different pressure conditions (0.1, 40, and 80 MPa) for 3 days. The mycelia were collected, frozen and ground in liquid nitrogen, and then resuspended in RNAiso Plus (Takara, Japan) and preserved at −80°C. The RNA extraction, transcriptomics sequencing and bioinformatics analysis were conducted by Novogene Bioinformatics Technology (Beijing, China). The methods associated with RNA extraction, transcriptomics sequencing and bioinformatics analysis were listed in the Supplementary materials. Analysis of differentially expressed genes (DEGs) was performed using DESeq2, v1.4.5^5^ (Anders and Huber, 2010) |log2 Fold-Change| ≥ 1 and padj ≤0.05 were set as criteria for the screening of DEGs between different groups. GO and KEGG enrichment analysis of annotated DEGs was implemented by the clusterProfiler R package.^6^

### RNA extraction and quantitative reverse transcription PCR (qRT-PCR)

To provide further confirmation of the DEGs, qRT-PCR was performed as described previously (Zhong et al., 2024). Total RNAs were extracted and used to synthesize cDNA using the RNAiso Plus (Takara, Japan) and PrimeScript RT reagent kit with gDNA Eraser (Perfect Real Time) (Takara, Japan), separately, according to the manufacturer’s protocols. The primer sequences used in the qRT-PCR were listed in Supplementary Table S2. The housekeeping gene *β*-actin was used as a reference gene. The relative expression level of targeted genes relative to the reference gene was determined using the 2^−∆∆Ct^ method (Livak and Schmittgen, 2001). The RNA-Seq fold changes were plotted against the qRT-PCR fold changes to calculate the correlation coefficients by taking the square root of the R-squared value (R^2^) (Wang et al., 2021).

### Statistical analysis

All experiments and groups were conducted at least in triplicate, with three biological replicates taken at each sample. All data were analyzed using SPSS Statistics 27 for Windows (SPSS Inc.).^7^ The differences between the variables were evaluated by one-way analysis of variance (ANOVA), followed by Tukey’s multiple comparison test. All results were expressed as mean ± standard error (SE) of the mean. Results were considered to be significant difference at the level of *p* < 0.05 level (*p < 0.05, **p < 0.01, ***p < 0.001). Graphs and statistical calculations were performed using GraphPad Prism 8 (GraphPad software, San Diego, CA).^8^ The histogram and pie chart were drawn using GraphPad Prism 8. The heatmap was drawn using TBtools software.^9^ The stacked bar plot was drawn using ChiPlot.^10^ The multi-group difference scatter plot was drawn using Omicshare tools.^11^

## Results

### Isolation and identification of hadal fungus

**Figure 1:**
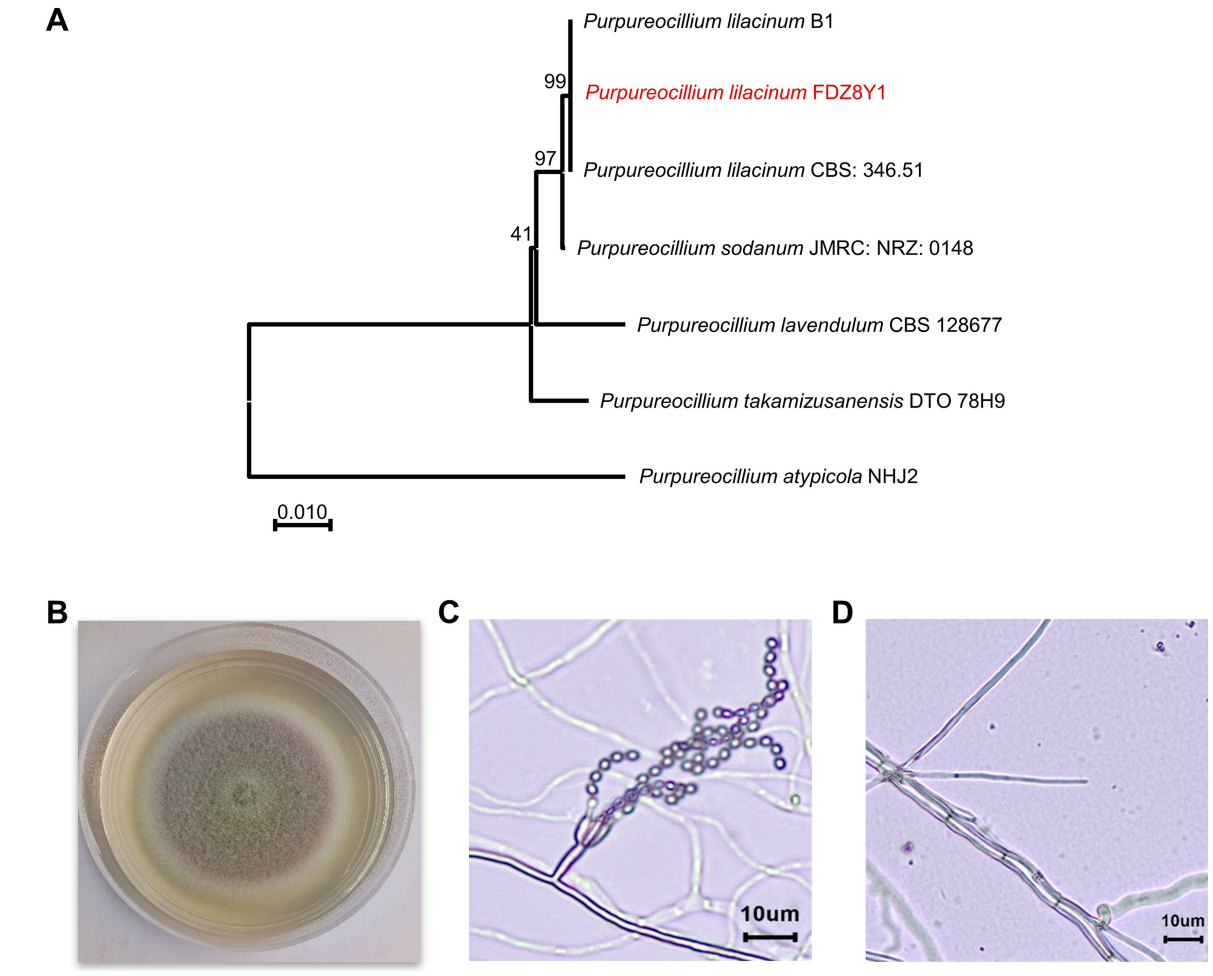
Identification of hadal-derived fungus *P. lilacinum* FDZ8Y1. (A) Neighbor-joining tree based on ITS gene sequences of *P. lilacinum* FDZ8Y1 (highlighted in red) and close taxa of the genus *Purpureocillium*. The numbers at the nodes indicate the bootstrap values. The scale bar represents substitutions per nucleotide base. (B) Colony morphology of *P. lilacinum* FDZ8Y1 on PDA medium after 7 days of culture at 28°C. (C, D) Micromorphological characterization of *P. lilacinum* FDZ8Y1 observed by microscope BX53 (Olympus Corporation, Japan).

A hadal strain, designated as FDZ8Y1, was isolated from 10,898 m sediment of the Mariana Trench and identified based on an analysis of the internally transcribed spacer (ITS) sequence and morphological characteristics. The phylogenetic tree was constructed using ITS sequences, which indicated that the isolated strain FDZ8Y1 belonged to *Purpureocillium lilacinum* (Figure 1A).

The morphological characteristics of *P. lilacinum* FDZ8Y1 on PDA medium were consistent with those previously described by Luangsa-Ard et al. (2011). The colonies initially exhibited a white coloration, which subsequently transitioned to purple after 3 days of incubation. The mycelium, which is white in color, encircled the pink conidia, which were located in the centre of the colony (Figure 1B). On PDA at 28°C for 7 days, the colonies exhibited a diameter of 36–42 mm. The micromorphology of *P. lilacinum* FDZ8Y1 showed typical conidiophores and phialides with chains of conidia (Figures 1C,D).

### *Purpureocillium lilacinum* FDZ8Y1 possesses considerable bioactive and nematicidal potential

**Figure 2:**
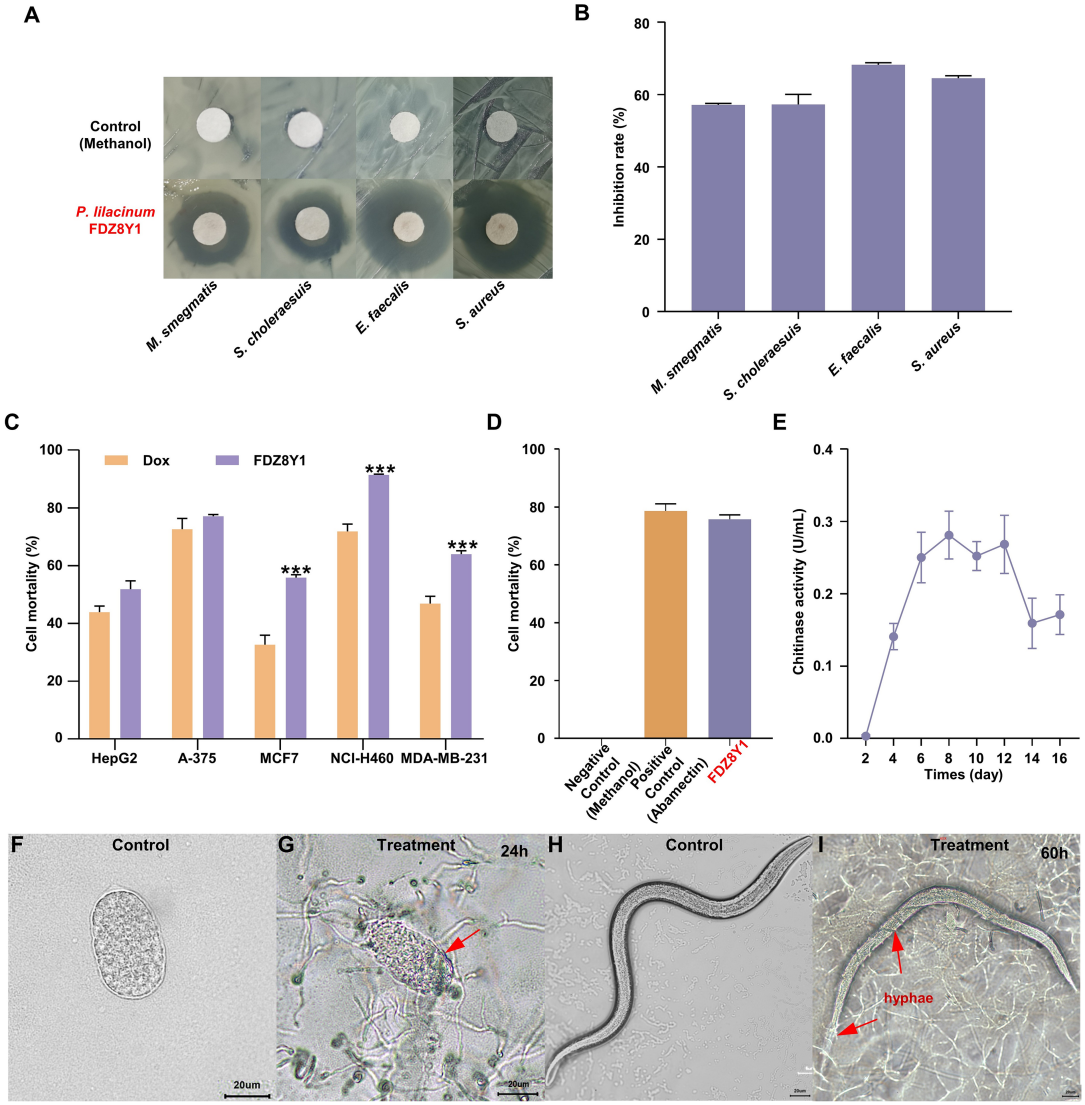
Characterization of hadal-derived fungus *P. lilacinum* FDZ8Y1. (A) Inhibition zones of secondary metabolites produced by *P. lilacinum* FDZ8Y1 (highlighted in red) detected by Kirby-Bauer method. The negative control was treated with an equal volume of methanol. (B) Inhibitory rates of secondary metabolites produced by *P. lilacinum* FDZ8Y1. (C) *In-vitro* cytotoxicity activity of secondary metabolites produced by *P. lilacinum* FDZ8Y1. (D) Nematicidal activity of secondary metabolites produced by *P. lilacinum* FDZ8Y1 (highlighted in red). (E) The time course of the chitinase activity of *P. lilacinum* FDZ8Y1. (F–I) Nematicidal ability was tested by nematode eggs and nematodes. Groups of control were untreated egg and nematode, respectively. The boundary of digested egg shell and hyphae entwined on the nematode were marked with red arrows.

To confirm the bioactive potential of the hadal sediment-derived fungus *P. lilacinum* FDZ8Y1, human pathogenic microorganisms and tumor cells were chosen to conduct the antibiotic assay. The crude extract was tested for antimicrobial activity toward seven pathogenic microorganisms using the disk diffusion method. The results showed the certain antimicrobial activity of *P. lilacinum* FDZ8Y1 against 4 pathogenic bacteria, *Mycobacterium smegmatis* MC2155, *Salmonella choleraesuis*, *Enterococcus faecalis* FA2-2, and *Staphylococcus aureus* ATCC25923, with inhibition rate of 57.1, 57.3, 68.3, and 64.5%, respectively (Figures 2A,B). The antitumor activities of the crude extract were evaluated using the MTT method with five tumor cell lines after 24 h. The results showed that the crude extract of FDZ8Y1 inhibited the growth of three cell lines significantly and the cell viability values at 500 μg/mL as follows: 91.4% (human lung cancer, NCI-H460), 64.0% (human breast carcinoma, MDA-MB-231), and 55.8% (human breast carcinoma, MCF-7) (Figure 2C), respectively.

The nematicidal activity of the crude extract was tested using *C. elegans*. It was found that fungal metabolites from *P. lilacinum* FDZ8Y1 showed anti-nematode activity (Figure 2D). It displayed 94.7% juvenile mortality after incubation for 24 h. The negative control methanol did not show any activity. UPLC-MS/MS analysis revealed that hadal-derived *P. lilacinum* FDZ8Y1 produced leucinostatins A (1218.6 g/mol) and leucinostatins B (1204.6 g/mol) (Supplementary Figure S1). From a biochemical perspective, enzymes produced by NF such as proteases, chitinases, and lipases constitute a powerful arsenal in the predatory mechanism (Freitas Soares et al., 2023). Furthermore, a series of DNS assays were conducted to quantify the amount of reducing sugars released from chitin to determine chitinase activity, which may be involved in the parasitic properties of the fungus. The time course of the chitinase activity of *P. lilacinum* FDZ8Y1 demonstrated that the highest activity occurred after approximately 8 days (Figure 2E).

The nematicidal bioassay of fungal mycelium showed that the eggshell of nematode eggs disappeared after coculture for 24–60 h. In comparison to the control (Figures 2F,G; Supplementary Figures S2A–D), the boundary of the egg shell started to be degraded and wrinkling (Figure 2G), the overall morphology of the egg swelled and buckled as penetration continued (Supplementary Figures S2B,C), then the fungi may have digested their contents. After 60 h of treatment, the hyphae exhibited a tendency to grow towards nematode eggs and adhere to surfaces (Supplementary Figure S2D). Additionally, the fungus FDZ8Y1 has the capacity to parasitize nematodes directly, thereby achieving insecticidal effects (Figures 2H,I; Supplementary Figures S2E–P).

These results indicated that hadal fungus *P. lilacinum* FDZ8Y1 displayed significant inhibitory activity against pathogenic bacteria and human cancer cell lines, suggesting that it may serve as a natural reservoir of bioactive SMs. In addition, *P. lilacinum* FDZ8Y1 exhibited a notable nematicidal capacity, suggesting its potential use as a biocontrol agent.

### High hydrostatic pressure (HHP) affects the growth, development, and production of SMs of *P. lilacinum* FDZ8Y1

In our previous studies, we found that HHP could affect the growth, development, and production of SMs of fungi (Peng et al., 2021; Deng et al., 2023). Here, we explored the impact of HHP on the growth, development, and SMs of the hadal fungus *P. lilacinum* FDZ8Y1 to identify the piezotolerance.

#### Piezotolerance and growth dynamics

**Figure 3:**
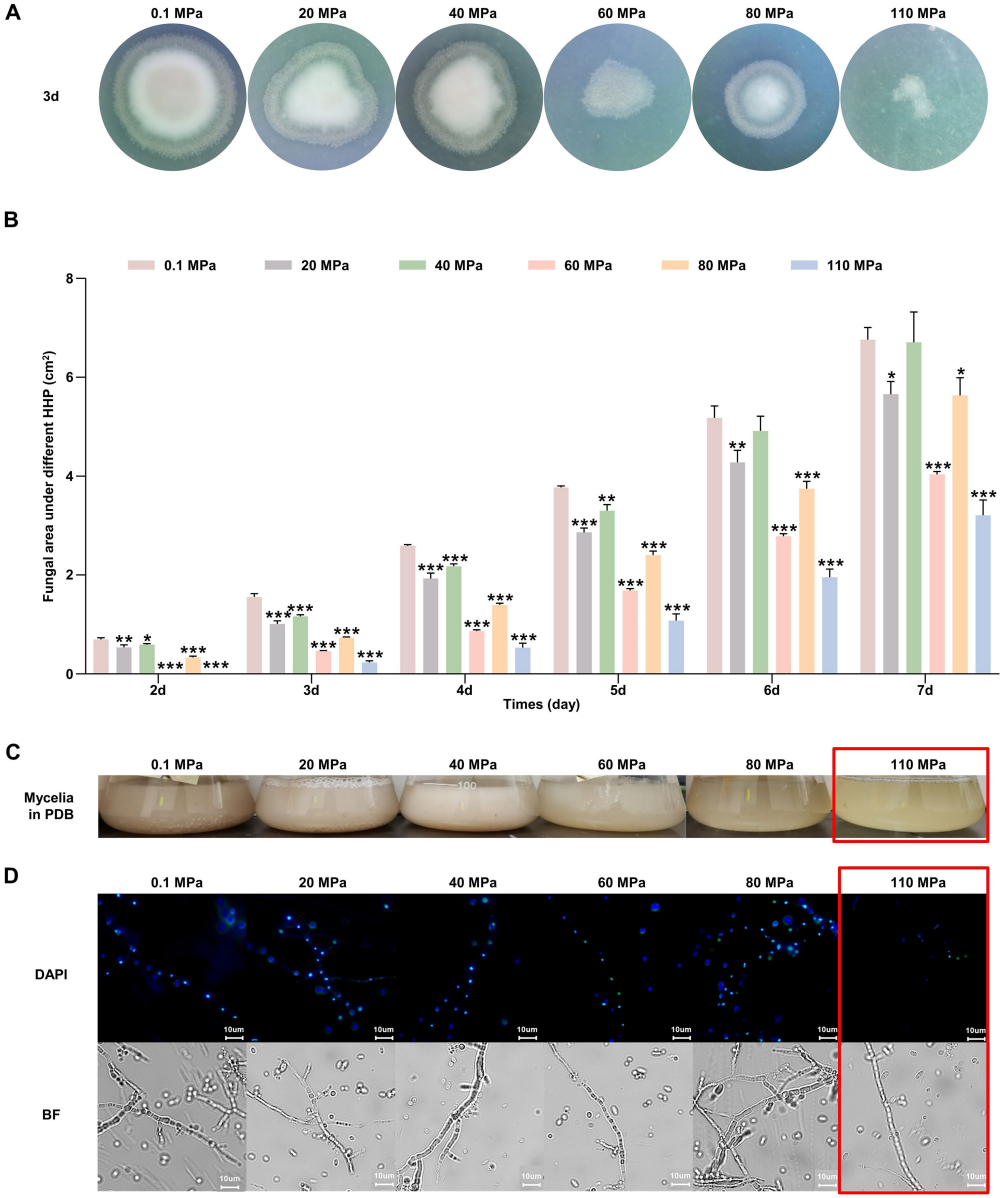
Effect of HHP on hadal-derived fungus *P. lilacinum* FDZ8Y1. (A) Colony phenotypes of spores treated with elevated HHP cultured on PDA for 3 days. (B) The colony area of *P. lilacinum* FDZ8Y1 cultured on PDA for 2–7 days after treating with different pressure. The unit of the area was cm^2^. (C) The vegetative growth statuses of mycelium exposed to elevated HHP on PDB. (D) HHP-treated mycelium blocks stained with DAPI to observe the nucleus. The 110 MPa-treated mycelium were framed in red box to highlight its poor piezotolerance. BF indicates bright field.

HHP assays demonstrated that spores of *P. lilacinum* FDZ8Y1 were able to germinate and grow after 14 days of incubation under pressures ranging from 20 to 110 MPa (Figure 3A). However, increased pressures significantly delayed spore germination and decreased spore vitality, with notable inhibition observed at 60, 80, and 110 MPa (Supplementary Figure S3). Then the colony areas and growth rates were calculated, which demonstrated the fungal development in further detail. The maximum growth rate of *P. lilacinum* FDZ8Y1 was observed at 0.1 MPa prior to 6 days of incubation, followed by 40 MPa, while the lowest growth rate was observed at 110 MPa. After 6 days, the fungal colony at 40 MPa exhibited the largest colony area, while the highest growth rate was observed at 80 MPa after 7 days (Figure 3B; Supplementary Figure S4). These results indicated that HHP imposes a stronger inhibitory effect on fungal growth during the early cultivation stages. HHP affects the germination and development of fungi derived from the Mariana Trench sediment.

#### Impact on vegetative growth

Fungal mycelium exposed to hydrostatic pressures ranging from 0.1 to 80 MPa could regrow in PDB. However, the mycelium exposed to 110 MPa experienced a loss of vitality and failed to recover its growth (Figure 3C). DAPI staining assay showed that hyphal nuclei were arranged in an orderly manner at hydrostatic pressures ranging from 0.1 to 80 MPa, but no stained nuclei were observed at 110 MPa, indicating cellular damage (Figure 3D). Thus, we speculated that spores of *P. lilacinum* FDZ8Y1 had a higher tolerance to HHP than that of hyphae, which contradicts the earlier opinion of Damare et al. (2008) that the fungal hyphae had a better resistance to HHP than fungal spores. The greater resistance of spores compared to mycelia can be attributed to their inherent structural and biochemical properties. Spores often have thick cell walls and protective coatings that shield them from environmental stressors. In contrast, mycelia, being metabolically active and more exposed, are less equipped to handle extreme conditions.

#### Effects on SMs production and antibacterial activity

**Figure 4:**
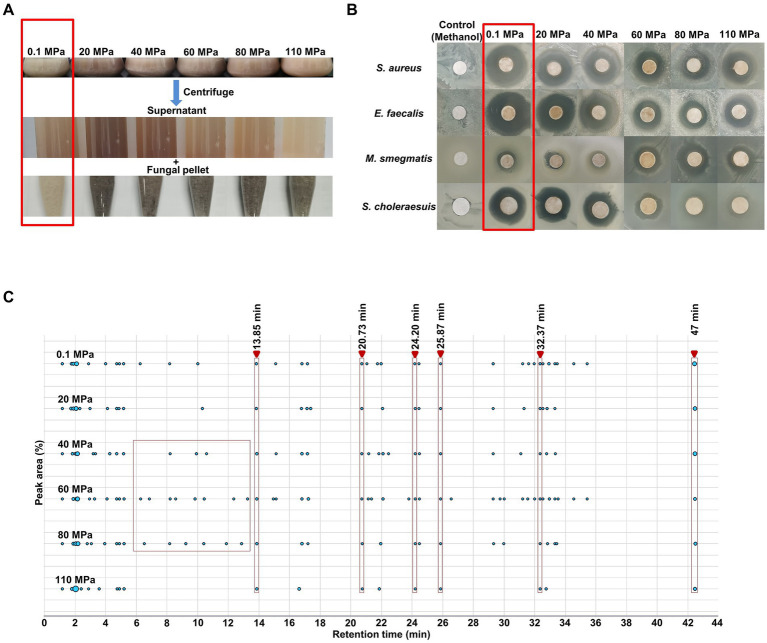
Effect of HHP on secondary metabolites of hadal-derived fungus *P. lilacinum* FDZ8Y1. (A) The changes of color in fermentation broths of spores cultured on PDB for 10 days after treated with elevated HHP. The fermentation broth and fungal pellets obtained from HHP-treated spores accumulated more pigments. (B) The antimicrobial activity of secondary metabolites produced by *P. lilacinum* FDZ8Y1 after treated with elevated HHP. The part highlighted by the red box represents the control group (0.1 MPa). (C) Peak area by HPLC of secondary metabolites produced by *P. lilacinum* FDZ8Y1 after treated with elevated HHP. The part highlighted by the red box represents the selected retention time.

HHP treatment resulted in noticeable changes in the pigmentation of fermentation broth, with darker coloration observed in fungal pellets obtained from HHP-treated spores at higher pressures (Figure 4A). Furthermore, the antibacterial activity of SMs was also influenced by HHP (Figure 4B). The results of the antibacterial activity showed HHP decreased the inhibition rates of SMs against *S. choleraesuis*, *E. faecalis* and *S. aureus*, particularly at pressures of 60, 80, and 110 MPa. The antibacterial activity of SMs against *M. smegmatis* was found to be significantly enhanced under 60, 80, and 110 MPa, with an inhibition rate of 51.3, 58.5 and 52.3%, respectively (Figure 4B; Supplementary Figure S5). HPLC analysis indicated that HHP treatment could altered the concentrations and diversity of compounds present in the SMs of *P. lilacinum* FDZ8Y1, with more peaks detected in the SMs of fungi incubated under pressures of 40, 60, and 80 MPa compared to 110 MPa (Figure 4C; Supplementary Figure S6). The contents of compounds, with a retention time (RT) of 13.85, 20.73, 24.20, 25.87, 32.37 and 42.47 min, respectively, were found to be altered under different hydrostatic pressures. These findings suggest that the application of HHP has an impact on the production and bioactivity of SMs of *P. lilacinum* FDZ8Y1.

The results highlight that HHP significantly impacts the growth dynamics, structural integrity, and SMs production of *P. lilacinum* FDZ8Y1. Spores exhibit higher pressure tolerance than hyphae, and the changes in SMs profiles and antibacterial activities under varying pressures underscore the adaptive biochemical responses of fungi to extreme environments. These findings deepen our understanding of fungal adaptation to deep-sea conditions and have implications for fungal preservation, biotechnological applications, and ecological studies of fungi in high-pressure environments.

### General structure of *P. lilacinum* FDZ8Y1 genome and comparison with other NF

In order to gain insight into the function traits at the genome level, the *P. lilacinum* FDZ8Y1 genome was sequenced and assembled into 35 contigs, with a total size of 36,255,754 bp (2,092,838 bp N50 contig length, 307x genome coverage), and a GC content of 59.12%. The longest contig length was of 4,657,805 bp. The genome of *P. lilacinum* FDZ8Y1 was predicted to have 8,319 protein-coding genes (Supplementary Figure S7A; Table 1). Subsequently, gene functional annotation was conducted. A total of 641, 120 and 1,834 genes were identified as encoding putative secreted proteins, CYP450 and PHI-associated genes, respectively (Table 1; Supplementary Figures S7B,C). A total of 2,492 (29.96%) protein-coding genes were predicted to display KOG functional annotation (Supplementary Figure S8).

**Table 1:** Genome features of the hadal-derived fungus *P. lilacinum* genome.

Features	Value
Genome Size (bp)	36,255,754
Coverage	307×
Number of reads	2,393,313
N50 Read Length (bp)	6,518
Number of Contigs	35
Max contig length (bp)	4,657,805
N50 contig length (bp)	2,092,838
GC content (%)	59.12
Repeat content (%)	2.09
Complete BUSCOs (%)	97.9
Number of protein-coding genes	8,319
Total length of coding sequences (Mb)	11.11
Average gene size (bp)	1,335
tRNA genes	96
Number of Secreted proteins	641
Number of CYP450 genes	120
Number of PHI genes	1,834
Number of CAZymes genes	385
Number of peptidase genes	115
Number of secondary metabolites clusters	43

*P. lilacinum* FDZ8Y1 possessed considerable antibacterial, antitumor and nematicidal activity as well as great enzyme activity. Consequently, a genome-based analysis was conducted on genes associated with enzymes and secondary metabolite synthesis, as well as genes related to transmembrane transporter proteins (Supplementary Figures S9, S10; Supplementary Table S5).

#### CAZymes and proteases

CAZymes are particularly interesting targets in the study of plant pathogens and nematode parasitism (Zerillo et al., 2013; Brouwer et al., 2014; Aranda-Martinez et al., 2016). Here, CAZymes analysis identified glycoside hydrolases (GH) as the most prevalent enzyme class, accounting for almost half of the identified enzymes (219) (Supplementary Figure S9A). The most abundant CAZyme family was GH18, which was represented by chitinases. These enzymes degraded the chitin present in the components of nematode cuticle and eggshell. Peptidases produced by fungi can be used for biological control of insect pests as well as various pathogens including bacteria, fungi, and nematodes (Semenova et al., 2020). The genome of *P. lilacinum* FDZ8Y1 exhibited a reduced number of fewer proteases (115 genes) than other egg-parasitic fungi (except for *Purpureocillium lavendulum*) (Supplementary Table S5). The most abundant family in *P. lilacinum* FDZ8Y1 was that of serine peptidase (36 genes), which is an important virulence factor in the process of NF invading nematodes (Supplementary Figure S9B). Hierarchical cluster analysis showed that CAZymes and proteases of hadal-derived *P. lilacinum* FDZ8Y1 exhibited similar patterns to those of terrestrial fungi, while the number of functional enzyme genes was lower (Supplementary Figure S9C), possibly indicating a lower demand for specific CAZymes and peptidase functions in the deep-sea environment.

#### Secondary metabolite biosynthetic genes

**Figure 5:**
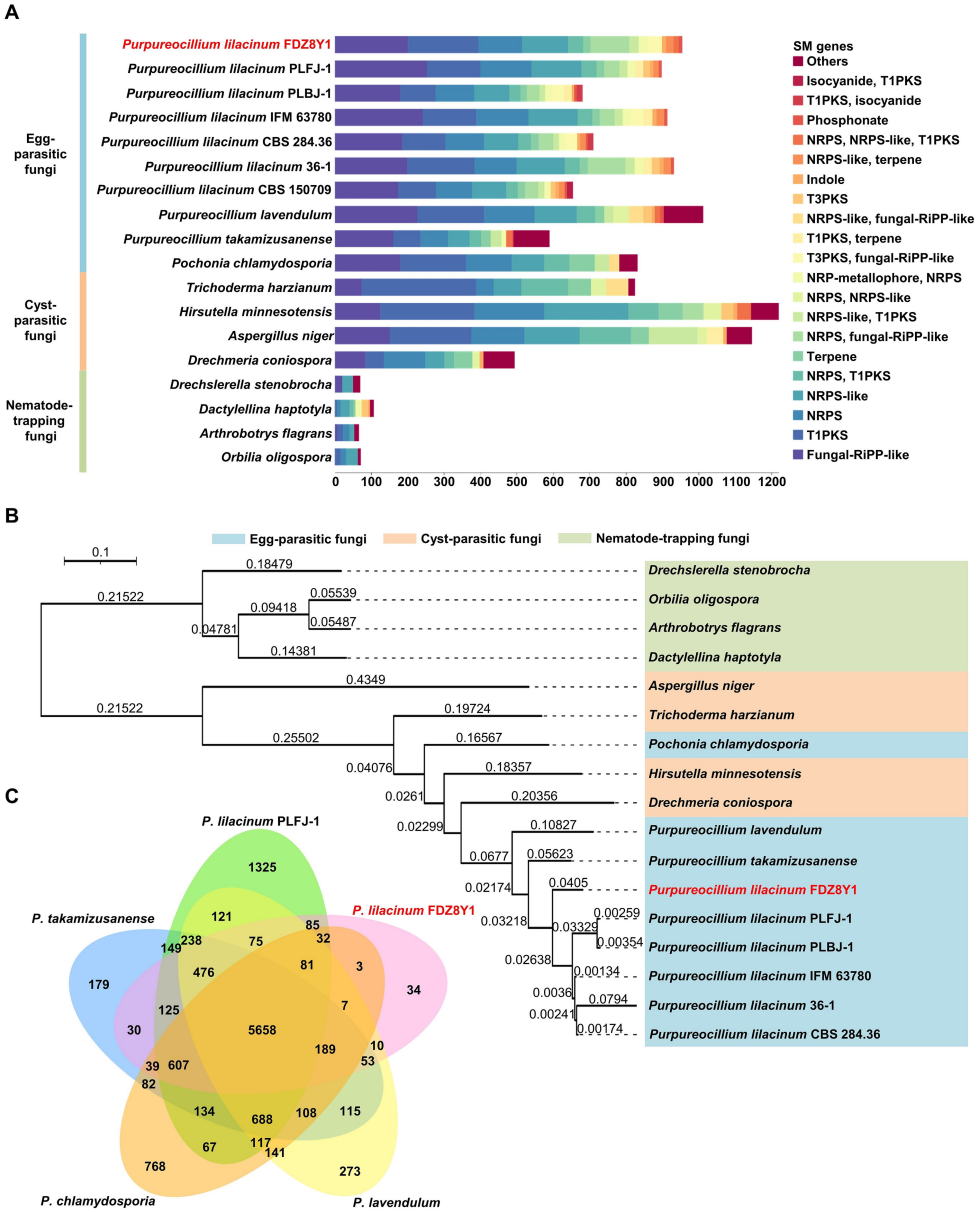
Comparative genomics and evolutionary analysis of hadal-derived fungus *P. lilacinum* FDZ8Y1. (A) Abundance of genes involved in secondary metabolite biosynthesis in *P. lilacinum* FDZ8Y1 (highlighted in red) and other selected fungal species. Bars represents the gene numbers for different kinds of secondary metabolite biosynthesis genes. (B) A phylogenomic tree constructed using single-copy orthologous proteins showing the evolutionary relationship of *P. lilacinum* FDZ8Y1 (highlighted in red) with other selected fungal species. Color codes for the three nematophagous fungi are shown in the figure. The branch lengths marked in branches indicate evolutionary distance between two species. (C) Numbers of orthologous clusters between five egg-parasitic fungi. A Venn diagram shows shared orthologous protein clusters among *P. lilacinum* FDZ8Y1 (highlighted in red), *P. lilacinum* PLFJ-1, *P. takamizusanense*, *P. lavendulum* and *P. chlamydosporia*.

To evaluate the capability of *P. lilacinum* FDZ8Y1 to produce SMs, the genome was annotated by antiSMASH to identify sequences containing biosynthetic gene clusters (BGCs). A total of 954 secondary metabolite biosynthetic genes (43 BGCs) were identified in FDZ8Y1 (Supplementary Figure S10A). These gene clusters included 10 Type I PKS, 6 NRPS, 7 fungal-RiPP-like clusters, 6 NRPS-like clusters, 3 terpene synthase clusters, 1 indole synthase clusters, 1 phosphonate synthase clusters, and 9 hybrid enzymes. In comparison to other sequenced species of egg-parasitic fungi, the number of BGCs in *P. lilacinum* FDZ8Y1 (43) was similar to that observed in *P. lilacinum* PLFJ-1 (45), PLBJ-1 (43), 36–1 (44), IFM_63780 (42), CBS_284.36 (47), CBS_150709 (45) and *P. lavendulum* (47), respectively, more than those in *Purpureocillium takamizusanense* (39), fewer than those in *Pochonia chlamydosporia* (50). Compared to cyst-parasitic fungi, the number of BGCs in *P. lilacinum* FDZ8Y1 was fewer than those in *Trichoderma harzianum* (50), *Aspergillus niger* (64) and *Hirsutella minnesotensis* (83). Compared to nematode-trapping fungi, the number of BGCs in *P. lilacinum* FDZ8Y1 was greater than that observed in *Drechslerella stenobrocha* (4), *Drechmeria haptotyla* (10), *Orbilia oligospora* (6) and *Arthrobotrys flagrans* (6) (Supplementary Figure S10B). Statistical analysis of genes on BGCs revealed that egg-parasitic fungi and cyst-parasitic fungi exhibited a greater number of secondary metabolite biosynthetic genes than nematode-trapping fungi (Figure 5A).

### Phylogenomic and orthologous analysis

A phylogenomic analysis was conducted to determine the relationship among *P. lilacinum* FDZ8Y1 and 16 other NF using single-copy orthologous proteins (Figure 5B; Supplementary Table S2). The results indicated that *P. lilacinum* FDZ8Y1 exhibited a closer relationship with egg-parasitic fungi and cyst-parasitic fungi. To annotate orthologous proteins, the protein-coding genes of *P. lilacinum* FDZ8Y1 were compared to those of other egg-parasitic fungi (Figure 5C). We found that among the total of 12,009 orthologous clusters, 9,430 were orthologous and 3,249 were single-copy gene clusters. A total of 5,658 clusters were found to be orthologous in all five fungi. Moreover, *P. lilacinum* FDZ8Y1 exhibited a greater number of lineage-specific gene clusters with *P. lilacinum* PLFJ-1 (85) than with *P. takamizusanense* (30), *P. lavendulum* (10) and *P. chlamydosporia* (3). The highest number of clusters shared between *P. lilacinum* FDZ8Y1 and PLFJ-1 reflected their close relationship. A total of 36 proteins (34 orthologous clusters) of *P. lilacinum* FDZ8Y1 had no orthologous proteins with any of the other four species. Among the 36 species-species genes, 13 could be annotated by eggnog-mapper, of which genes with functions of transmembrane transporter and the synthesis of SMs were identified (Supplementary Table S6), including sugar transporter, oligopeptide transporter, major facilitator superfamily protein and polyketide synthase. It can be speculated that unique transporter genes and SM biosynthesis genes may be the key factors enabling *P. lilacinum* FDZ8Y1 to cope with the extreme hadal environments.

### Metabolic potentials of *P. lilacinum* FDZ8Y1

**Figure 6:**
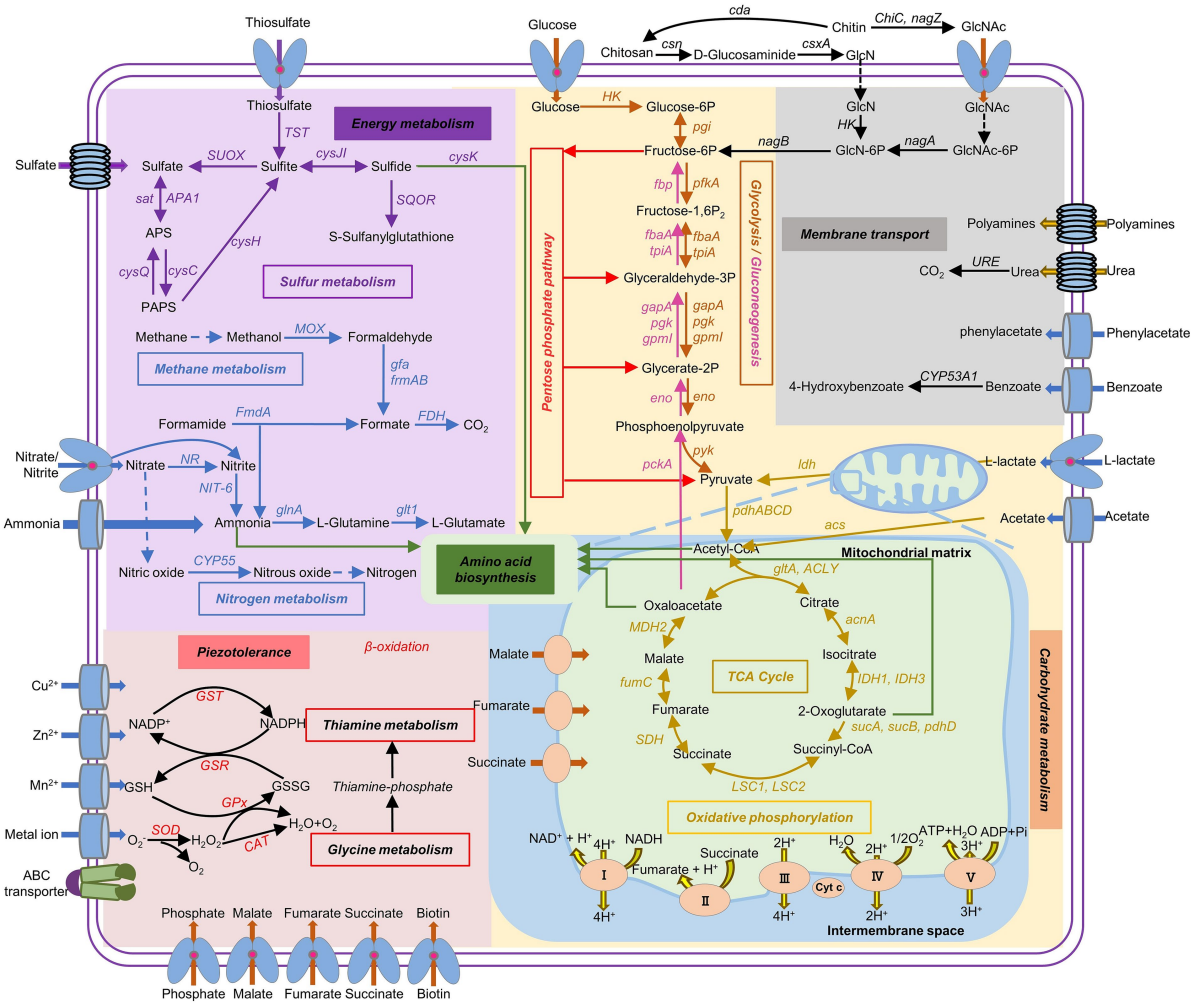
Metabolic potential predicted from genomic information of hadal-derived fungus *P. lilacinum* FDZ8Y1. The pathways with all of the genes identified are shown in solid arrows, while the pathways missing key genes are shown in dash arrows. Gene names and the proteins they encode were listed in Supplementary Table S7.

Based on the KEGG database, multiple energy production pathways and genes related to stress resistance were predicted in the genome of strain FDZ8Y1 (Figure 6; Supplementary Table S7). Detected genes included those involved in glycolysis/gluconeogenesis pathways (25), TCA cycle (21), the pentose phosphate pathway (19), pyruvate metabolism (28), and oxidative phosphorylation (65), almost completing the carbohydrate-metabolizing pathways. Furthermore, genes involved in nitrogen metabolism (13) and sulfur metabolism (14) were identified. The genome of *P. lilacinum* FDZ8Y1 possessed the complete pathways for assimilatory nitrate / sulfate reduction, indicating that hadal fungi could utilize nitrate and sulfate as electron acceptors for energy production under anaerobic conditions (Chen et al., 2021). Finally, genes related to stress resistance were also predicted, including those encoding enzymes for peroxisomes, the antioxidant system (SOD, CAT, GPx, GR and GST), fatty-acid oxidation (*α*- and *β*-oxidation), and other oxidant-related enzymes. Additionally, genes involved in glutathione and thiamine metabolism, as well as those related to compatible solutes (such as glycine, glutamate, and betaine), were predicted in the genome of the strain FDZ8Y1.

### Transcriptome overview, differentially expressed genes (DEGs) and enrichment analysis

In order to elucidate the molecular mechanism of piezotolerance, RNA sequencing analysis was conducted on *P. lilacinum* FDZ8Y1 under different hydrostatic pressures (0.1, 40 and 80 MPa). Raw reads information was listed in Supplementary Table S8, which indicated the reliability of RNA-seq data in this work. Principal component analysis (PCA) was performed on the gene expression values (FPKM) of all samples, and the results showed that intergroup samples exhibited distinct characteristics (Supplementary Figure S11A). A total of 3,360 and 4,193 DEGs (|log2 Fold-Change| ≥ 1 and padj ≤0.05) were observed in *P. lilacinum* FDZ8Y1 under 40 MPa and 80 MPa, respectively (Supplementary Figures S11B–D).

KEGG and GO enrichment analysis of DEGs were conducted, and the top 20 terms were selected for further enrichment (Supplementary Figure S12). These results implied that components of membrane, biosynthesis of secondary metabolites and amino acids, carbohydrate metabolism and oxidation–reduction process play an essential role in the response to the HHP condition. All the selected DEGs identified by qRT-PCR exhibited comparable expression patterns to those observed in the RNA-seq data, which demonstrated the reliability of the RNA-seq data (Supplementary Figure S13).

### Gene expression patterns of *P. lilacinum* FDZ8Y1 in response to hydrostatic pressure

**Figure 7:**
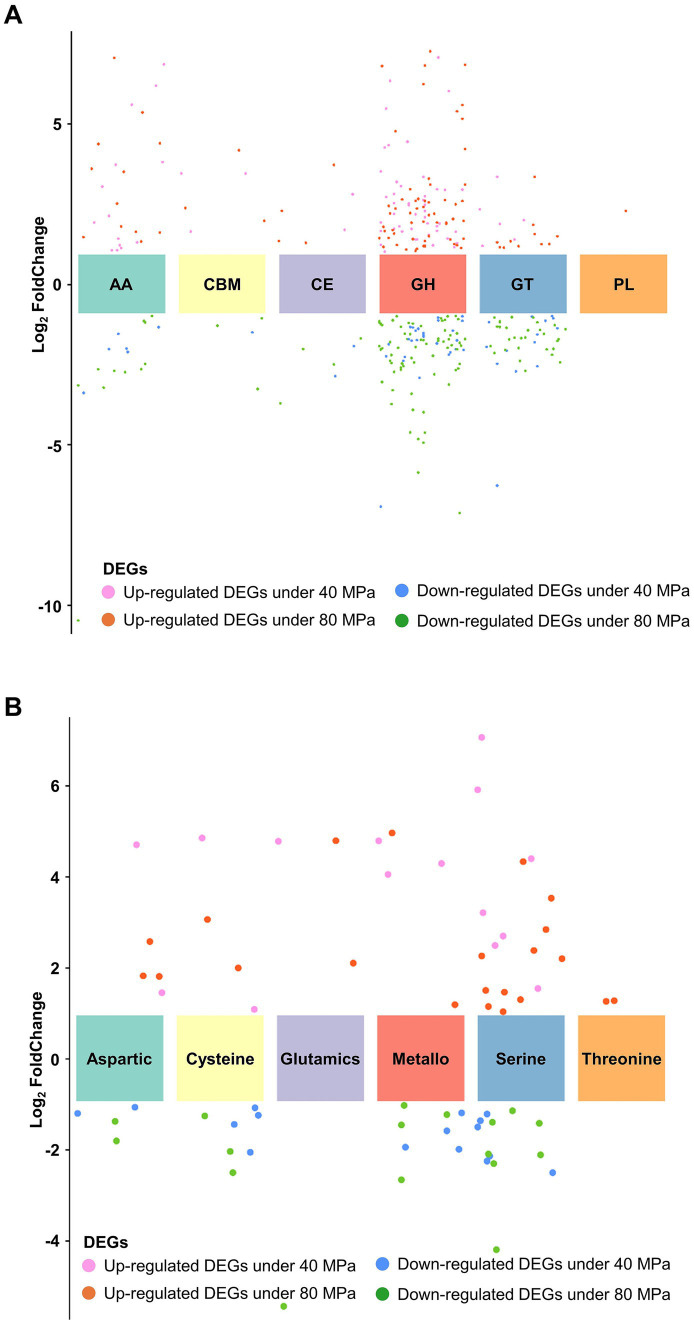
Expression patterns of enzyme families in *P. lilacinum* FDZ8Y1. (A) Expression profile of different CAZyme families. (B) Expression profile of different peptidase families.

Based on genomic analysis and enrichment analysis of DEGs, the related gene expression profiles were subjected to analysis to gain insight into the underlying molecular mechanism of HHP tolerance. Results showed that 233 genes involved in CAZymes were significantly regulated under HHP conditions, with 115 genes upregulated, including 51 secretory proteins (Figure 7A). Among these, genes related to GH, involved in degrading extracellular glycogen and other substrates (GH18, GH2, GH105, GH24/25, GH5), were significantly upregulated (log2 fold change >1) (Supplementary Table S9). Additionally, 28 peptidases were significantly upregulated in response to HHP, with 14 being secretory proteins (Figure 7B). Notably, serine proteases, including 5 subtilisins (S08), signal peptidases (S26), carboxypeptidase (S10) and aorsin (S53) were upregulated under HHP conditions (Supplementary Table S10), suggesting that fungi may secrete enzymes to degrade extracellular substances to obtain energy. Nine key genes involved in biosynthesis of SMs were significantly regulated under HHP. These include PKS genes (*gloL*, *nscA*, *ustP*, *csyA*, *lcsB*) and a NRPS (*lcsA*) gene, which linked to the biosynthesis of compounds such as pneumocandins, neosartoricin B, ustilaginoidins, 3,5-dihydroxybenzoic acid, and leucinostatins, exhibiting a broad spectrum of biological activities (Arai et al., 1973; Chen et al., 2013; Chen and Hu, 2022; Seshime et al., 2010; Xu et al., 2021; Yin et al., 2013) (Supplementary Table S11).

The application of HHP resulted in the upregulation of several genes involved in cell wall structure, lipid and fatty acid biosynthesis (Supplementary Table S12). However, most genes related to glycolysis/gluconeogenesis, the TCA cycle, and oxidative phosphorylation were downregulated under HHP conditions, except for a few genes. Additionally, most genes related to amino acid synthesis were downregulated, except for glutarate-semialdehyde dehydrogenase gene (*gabD*) and argininosuccinate synthase gene (*argG*), which were upregulated in response to 40 MPa and 80 MPa, respectively, indicating that a different energy supply and survival mechanism under HHP (Luo et al., 2020).

Genes related to ROS metabolism and glutathione metabolism were upregulated under HHP, suggesting an antioxidant defense mechanism to prevent oxidative stress. Oxidative stress has been implicated as a key mediator of heat shock protein (HSP) induction. Genes related to HSPs (HSP30, HSP70, HSP90,) and molecular chaperone (DnaJ) were upregulated under HHP (Supplementary Table S12), indicating that heat shock factor can be activated by oxidative stress to increase the synthesis of protective HSPs (Kovács et al., 2019; Yan et al., 2002; Yusof et al., 2021; Yusof et al., 2022; Yusof et al., 2024).

MAPK signaling pathway respond to environmental stress by initiating signaling cascades that lead to various cellular responses. In this study, genes associated with mitogen-activated protein kinase kinase (MAPKK, MKK1,2 and STE7) and mitogen-activated protein kinase (MAPK, MAF1) were activated under different pressure (Supplementary Table S12). Cell cycle regulation is also a critical function that is essential for stress adaptation. Here, the expression of *hog1* and *sho1* were downregulated, while the expression of genes involved in cell cycle arrest and DNA replication were upregulated under HHP. The HOG-MAPK signaling pathway regulated multiple stages of the cell cycle by acting on core components of the cell cycle machinery (de Nadal and Posas, 2022). Thus, it was postulated that a delay in the cell cycle was necessary for cells to generate adaptive responses before progressing into the subsequent phase of the cycle.

## Discussion

The contribution of fungi to terrestrial ecosystem processes is well documented, but aspects of the marine ecosystem remain underexplored. Recent studies suggested that fungi interact with other halobios (O’Rorke et al., 2014; Yarden, 2014) and may play a key role in nutrient cycling of deep-sea sediments (Orsi et al., 2013). Natural products derived from the deep sea are important and novel due to their unique chemical structures and bioactivities. However, their development has been limited because of the challenging and costly nature of deep-sea exploration and extraction, and our understanding of the functions of hadal fungi in deep-sea ecosystems remains to be investigated. In our study, we isolated a filamentous fungus, *P. lilacinum* FDZ8Y1, from the Mariana Trench sediment, which exhibited antibacterial, antitumor and nematicidal activity. *P. lilacinum* is widely known as a biological control agent against plant parasitic nematodes via the production of toxic compounds, enzymes and parasitism (Satou et al., 2008). The mycelium of *P. lilacinum* could invade the epidermis of the nematode directly and produce adherent spores on the surface, which are adsorbed and then infest the nematode (Sosa et al., 2018). Thus, how this fungus is able to tolerate HHP conditions and the biological functions of its secondary metabolites stimulate our interest.

Deep-sea environments, characterized by their unique and extreme conditions, represent an underexplored reservoir of novel natural products with distinctive structural and biological properties (Arifeen et al., 2019; Arifeen et al., 2020). These environments drive the evolution of unique metabolic pathways in deep-sea organisms, potentially leading to the production of new and bioactive SMs (Dong et al., 2024; Sun et al., 2020; Zhao et al., 2022). In our study, *in vitro* biochemical tests demonstrated that hadal-derived *P. lilacinum* FDZ8Y1 has the capacity to produce compounds with significant biological activity. Traditionally, the discovery of novel SMs has relied on terrestrial sources and shallow-sea sources, but the depletion of these resources and the redundancy of known compounds pose challenges (Bills and Gloer, 2016; Petersen et al., 2020). Our results demonstrated that deep-sea-derived fungi have a higher potential for yielding novel compounds with unique bioactivities that can address unmet medical needs, including the fight against drug-resistant pathogens. Increasing evidence suggests that ecological interactions may regulate SM production (Drott et al., 2017). Our previous studies have demonstrated that HHP can influence the biosynthesis of fungal SMs by regulating the expression of PKS genes (Deng et al., 2023; Peng et al., 2023). In this study, fungi under HHP conditions produced more melanin and other compounds, as well as changes in their biological activities. By subjecting the hadal-derived fungus to varying HHP conditions, we effectively mimicked its natural environment, thereby activating stress-responsive biosynthetic pathways that remain silent under standard laboratory conditions. Our findings underscore the potential of HHP as a powerful tool for discovering previously unrecognized bioactive compounds. The bioactive natural products produced by *P. lilacinum* FDZ8Y1 provide evidence for fungi-ecology interactions, particularly with nematodes and microbes. Among the significant compounds identified is leucinostatins (Supplementary Figure S1), a family of lipopeptide antibiotics isolated from *Purpureocillium* genus. Known for its antimicrobial and antitumor properties, leucinostatins disrupt membrane phospholipids, leading to membrane damage. Exploring deep-sea organisms with novel cultivation methods like HHP stimulation offers a promising approach to discovering new bioactive compounds. This strategy leverages the unique adaptations of deep-sea fungi to uncover novel compounds for pharmaceuticals, agriculture, and biotechnology.

Fungi are thought to have a relatively high tolerance to hydrocarbons, and they are likely primary degraders of organic carbon via secreted extracellular enzymes (Al-Nasrawi, 2012; McGenity et al., 2012). Recently, metatranscriptomic analyses confirmed the active participation of fungi in carbon cycling by detecting the fungal CAZyme transcripts (Chrismas and Cunliffe, 2020; Baltar et al., 2021). Functional analysis in *P. lilacinum* FDZ8Y1 showed that the CAZymes are dominated by GHs, GTs and AAs, which is consistent with the proportions of CAZymes in pelagic fungi (Baltar et al., 2021). These results suggest different ecological and biogeochemical roles for fungi and prokaryotes in the processing of carbohydrates (Zhao et al., 2020). *P. lilacinum* FDZ8Y1 also had a certain number of genes involved in cellulose and hemicellulose degradation, including GH and AA, which are essential for the degradation of polysaccharides and oxidative degradation of cellulose and chitin (Lombard et al., 2014). Furthermore, transcriptomic analyses also showed that genes involved in GHs, GTs and AAs were highly expressed under HHP conditions compared to CEs and CBMs. Among these genes, chitinase (GH18) was the most abundant and upregulated CAZymes, which is essential for the degradation of chitin, the most abundant renewable polymer in the oceans and an important source of carbon and nitrogen for marine organisms, making the process of chitin degradation as a key step in the marine nutrient cycle (Souza et al., 2011). Based on these findings, it is evident that *P. lilacinum* FDZ8Y1 plays a significant role in marine carbon cycling and nutrient dynamics, particularly through the degradation of chitin and other polysaccharides under HHP.

Although carbohydrates are the main components of macromolecules in marine organisms (~10%), proteins form the major part of the organic matter of marine plankton cells (>50%) (Hedges et al., 2002). Fungal degradation of proteins in global ocean was recently evaluated by analyzing fungal expression level of peptidases and CAZymes in global metagenomic and metatranscriptomic data (Breyer et al., 2022). In this study, the most abundant proteases in *P. lilacinum* FDZ8Y1 were serine peptidases and metallo peptidases, while cysteine peptidases were the third most abundant type of protease, as reported previously for pelagic fungi (Zhao et al., 2020; Breyer et al., 2022). Transcriptomic data showed that serine proteases were the main proteases utilized by *P. lilacinum* FDZ8Y1 under HHP conditions. Additionally, subtilisin (S08), a subfamily reported to be involved in nutrition and mostly secreted from the cell (Rawlings et al., 2018), was widely expressed. The patterns in the total gene abundance, diversity, expression and the percentage of peptidases and CAZymes from *P. lilacinum* FDZ8Y1 demonstrated that hadal-derived fungi participated in the carbon cycle (carbohydrates) and nitrogen cycle (proteins), highlighting potentially ecological roles in the deep sea.

Transporters are essential for nutrient acquisition, cellular homeostasis, and stress responses. They mediate the exchange of chemicals and signals inside and outside the cell membrane. Most transporters were found to exhibit resistance to drugs and stress (Costa et al., 2014; Johns et al., 2021). Active membrane transporters can catalyze transport across a membrane by coupling solute movement to a source of energy such as ATP and substances (Shilton, 2015). In this study, genes involved in MFS superfamily, APC superfamily and ABC superfamily were activated to transfer nutrition and amino acid (Supplementary Table S13). Amino acid transporters are membrane transport proteins that mediate the movement of amino acids in and out of cells or organelles, which are involved in many important physiological functions, including nutrient supply, metabolic transformation, energy homeostasis, and redox regulation (Xia et al., 2024). Under HHP conditions, fungi may activate these transporters to directly transfer amino acids to intracellular to conserve energies. Although the intrinsic mechanism of metabolic pathways in response to environmental stress remains unknown, we believed that the upregulated genes related to secretory proteins and transmembrane transport proteins provide a non-self-synthesized energy supply for hadal fungi under HHP conditions to save energy.

In extreme environments, such as the deep sea with HHP, cells suffer from an imbalance in oxidation and reduction (Xiao and Zhang, 2014), which can cause cellular damage (Poljsak et al., 2013). In previous studies, when exposed to HHP conditions, hadal fungi faced the important challenge of responding to oxidative stress (Li et al., 2022; Zhong et al., 2024). The antioxidant defense system mainly includes antioxidant enzymes and antioxidant complexes, among which SOD is one of the key antioxidant enzymes (Ighodaro and Akinloye, 2018). In our study, the genes related to SOD were upregulated under HHP conditions, which directly proves the connection between the antioxidant defense mechanism and microbial tolerance to HHP. We believe that *P. lilacinum* FDZ8Y1 may activate the antioxidant defense system to cope with HHP in the deep-sea extreme environments.

**Figure 8:**
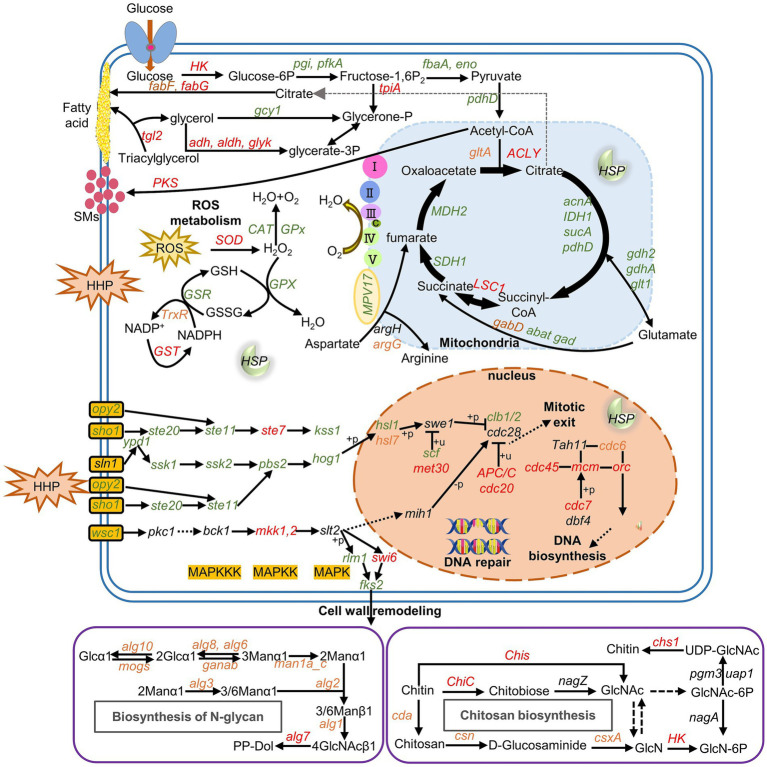
Gene expression pattern of *P. lilacinum* FDZ8Y1 regulated by HHP the pathways with all of the genes identified are shown in solid arrows, while the pathways missing key genes are shown in dash arrows. Red, green and black-labeled genes indicate genes upregulated, downregulated, and not significantly expressed under HHP conditions, respectively. Orange-labeled genes indicate different expressed under HHP conditions (40 MPa and 80 MPa). Gene names and the proteins they encode were listed in Supplementary Table S7.

Finally, a diagram of gene expression patterns regulated by HHP in the strain *P. lilacinum* FDZ8Y1 was generated (Figure 8; Supplementary Table S7). Firstly, the HOG-dependent MAPK pathway regulates the cell cycle arrest, giving the cells a buffer time to adapt to the HHP. Secondly, the SLT2-dependent MAPK pathway regulates the cell wall integrity, enabling the cells to resist the external stressors. Notably, pathways of glycolysis/gluconeogenesis, the TCA cycle, and oxidative phosphorylation are silenced to minimize ROS generation. Concurrently, fatty acid metabolism supports cell wall integrity and energy production. Secretory enzymes and transporters are also activated to obtain the energy needed under HHP conditions. Furthermore, genes related to antioxidant defense system and heat shock proteins are also detected to reduce ROS generation and increasing ROS removal in cells.

## Conclusion

In this study, we isolated a nematophagous and piezotolerant strain, *P. lilacinum* FDZ8Y1, from the Mariana Trench sediment. The strain exhibited significantly nematicidal activity and adaptation to high hydrostatic pressure. Moreover, the fungal natural products displayed a broad spectrum of biological activities. Hadal-derived *P. lilacinum* possessed abundant secondary metabolite biosynthesis gene clusters, indicating its potential to synthesize novel bioactive natural products. Genomic data also indicated that the strain had broad potential in carbon, nitrogen, and sulfur metabolism. Transcriptomic analysis demonstrated that hadal fungi had unique regulation mechanism to extreme environments. This is the first report on nematicidal fungi from deep-sea ecosystems and their adaptation to HHP. This study not only provides new candidates for bioactive compounds and entomopathogenic fungi, offering a new approach for developing deep-sea biological resources, but also explores the pressure adaptation mechanisms of filamentous fungi in extreme environments, laying a foundation for studying their ecological functions.

## Data Availability

The datasets presented in this study can be found in online repositories. The names of the repository/repositories and accession number(s) can be found in the article/Supplementary material.

## Footnotes

^1^https://www.ncbi.nlm.nih.gov/

^2^https://github.com/davidemms/OrthoFinder

^3^https://mafft.cbrc.jp/alignment/software/

^4^https://itol.embl.de/

^5^https://github.com/thelovelab/DESeq2

^6^https://www.rdocumentation.org/packages/clusterProfiler/versions/3.0.4

^7^https://www.ibm.com/support/pages/downloading-ibm-spss-statistics-27

^8^https://www.graphpad.com/scientific-software/prism/www.graphpad.com/scientific-software/prism/

^9^https://github.com/CJ-Chen/TBtools-II/releases

^10^https://www.chiplot.online/

^11^https://www.omicshare.com/tools/
