# Supplementary material for: Revealing the metabolic potential and environmental adaptation of nematophagous fungus, Purpureocillium lilacinum, derived from hadal sediment
Source: Front Microbiol. 2024 Nov 6;15:1474180. doi: 10.3389/fmicb.2024.1474180 (PMC11576294; doi:10.3389/fmicb.2024.1474180)
Supplement: Supplementary file 1 [file Data_Sheet_1.pdf]

# Revealing the metabolic potential and environmental adaptation of nematophagous fungus, *Purpureocillium lilacinum*, derived from hadal sediment

Yongqi Li[1], Changhao Zhang[1], Maosheng Zhong[1], Shenao Hu[1], Yukun Cui[1], Jiasong Fang[1, 2*], and Xi Yu[1*]

**Address**: [1]Shanghai Engineering Research Center of Hadal Science and Technology, College of Oceanography and Ecological Science, Shanghai Ocean University, Shanghai, 201306, China

[2]Laboratory for Marine Biology and Biotechnology，Qingdao Marine Science and Technology Center, Qingdao, China.

*Corresponding authors: JF (jsfang@shou.edu.cn), XY (xyu@shou.edu.cn);

**Key words:** Piezotolerance, hadal fungi, Mariana Trench, adaptation mechanism, transcriptome

**This document includes:**

Supplementary Materials and Methods

Supplementary Figures S1 to S13

Supplementary References

**Supplementary Materials and Methods**

**Genomic DNA assembly and component prediction**

The low-quality of genome of *P. lilacinum* FDZ8Y1 reads were filtered by the SMRT Link v8.0 (https://www.pacb.com/smrt-link/), then the filtered reads were assembled using software Falcon (https://github.com/falconry/falcon) to generate contigs (Wick et al., 2017; Reiner et al., 2018). Racon (version 1.4.1) (https://github.com/lbcb-sci/racon) and pilon (version 1.22) (https://github.com/broadinstitute/pilon) were used for sequence correction (Walker et al., 2014). Benchmarking Universal Single-Copy Orthologs (BUSCO) assessment was used to assess the integrity of assembled genome.

Genome component prediction included the prediction of the coding gene, repetitive sequences and non-coding RNA. *ab initio* prediction was performed using Augustus (version 2.7) (https://github.com/Gaius-Augustus/Augustus) (Stanke et al., 2008) to retrieve the related coding sequence (CDS). The interspersed repetitive sequences, tandem repeat sequences, transfer RNA (tRNA) and ribosomal RNA (rRNA) were predicted using RepeatMasker (Version open-4.0.5) ( https://www.repeatmasker.org/RepeatMasker/) (Saha et al., 2008), Tandem Repeats Finder (TRF, Version 4.07b) (https://github.com/Benson-Genomics-Lab/TRF) (Benson, 1999), tRNAscan-SE (Version 1.3.1) (Lowe and Eddy, 1997) (https://github.com/UCSC-LoweLab/tRNAscan-SE) and rRNAmmer (http://www.cbs.dtu.dk/services/RNAmmer/) (Lagesen et al., 2007) software,

respectively.

**Genome characterization and function annotation**

All obtained CDSs were annotated from NCBI non-redundant protein database (NR) (https://www.ncbi.nlm.nih.gov/refseq/about/nonredundantproteins/) (Li et al., 2002), Protein family (Pfam) (http://pfam.xfam.org/) (Finn et al., 2014), Clusters of orthologous groups for eukaryotic complete genomes (KOG) (https://ftp.ncbi.nlm.nih.gov/pub/COG/KOG/) (Koonin et al., 2004), Swiss-Prot (https://www.sib.swiss/swiss-prot), Kyoto Encyclopedia of Genes and Genomes (KEGG) (https://www.genome.jp/kegg/) (Kanehisa et al., 2008), and Gene Ontology (GO) (https://www.geneontology.org/) (Ashburner et al., 2000). Transporter Classification Database (TCDB) (https://www.tcdb.org/) (Saier et al., 2021) and Cytochromes P450 (CYP450) (https://drnelson.uthsc.edu/) (Kohler et al., 2015) were identified using Diamond (version v2.1.8) (https://github.com/bbuchfink/diamond) (e-value of 1e-5, coverage $\geq$ 0.4) (Buchfink et al., 2021). The proteases were identified by the MEROPS peptidase database (https://www.ebi.ac.uk/merops/), using Diamond (e-value of 1e-10) (Rawlings et al., 2002). The carbohydrate-active enzymes (CAZymes) were predicted using the dbCAN3 Database (http://aca.unl.edu/dbCAN3/index.php) (Zheng et al., 2023) with HMMER dbCAN (e-value $<$ 1e-15, coverage $>$ 0.35). The secondary metabolite genes were identified through the online antiSMASH database (https://fungismash.secondarymetabolites.org/#!/start) (Blin et al., 2023).

**RNA extraction, transcriptomics sequencing and bioinformatics analysis**

**i RNA extraction.** Total RNAs were extracted using the TRIzol Reagent (Invitrogen, CA, USA) according to the manufacturer's protocol. RNA integrity was assessed using the RNA Nano 6000 Assay Kit of the Bioanalyzer 2100 system (Agilent Technologies, CA, USA).

**ii Transcriptome sequencing.** Total RNA was used as input material for the RNA sample preparations. Finally, samples with RNA integrity number (RIN) values greater than 8 were used for library construction. Sequencing libraries were generated using NEBNext® Ultra™ RNA Library Prep Kit for Illumina® (NEB, USA) following manufacturer's recommendations, and index codes were added to attribute sequences to each sample. Oligo (dT) magnetic beads were used to enrich mRNA with PolyA tails, and then the mRNA was randomly fragmented by divalent cations in the NEB fragmentation buffer. The first and second strands of cDNA were synthesized using the fragmented mRNA as template and random oligonucleotides as primers. To screen out 370-420 bp cDNA fragments, the library fragments were purified using the AMPure XP system (Beckman Coulter, Beverly, USA). The screened cDNA was amplified by PCR, and the PCR product was purified using AMPure XP beads. Finally, the library was constructed. The library quality was assessed using Qubit 2.0 fluorometer, and then Agilent 2100 Bioanalyzer was used to detect the insert size of the library. After the expected insert size was verified, quantitative reverse-transcriptase PCR (qRT-PCR) was used to accurately quantify the effective concentration of the library to ensure the quality of the library. According to the manufacturer's instructions, TruSeq PE Cluster

Kit v3-cBot-HS (Illumina) was used to cluster the index-coded samples under the cBot Cluster Generation System. After clustering, the library was sequenced on Illumina Novaseq platform, and 150 bp paired-end reads were generated.

**iii Bioinformatics analysis.** Raw reads in fastq format mainly contained the sequence information of sequencing fragments and their corresponding sequencing quality information. In this step, clean reads were obtained by removing reads containing adapter sequences, reads containing poly-N, and low-quality reads from the raw data. Furthermore, Q20, Q30, and GC content of the clean data were calculated. All the subsequent analyses were based on clean data with high quality. Index of the reference genome was built using Hisat2 v2.0.5 (https://daehwankimlab.github.io/hisat2/) and paired-end clean reads were aligned to the reference genome using Hisat2 v2.0.5. We selected Hisat2 as the mapping tool for that Hisat2 can generate a database of splice junctions based on the gene model annotation file and thus a better mapping result than other non-splice mapping tools (Mortazavi et al., 2008). FeatureCounts v1.5.0-p3 (https://subread.sourceforge.net/) was used to count the reads numbers mapped to each gene (Liao et al., 2014). And then fragments per kilobase of exon model per million mapped reads (FPKM) of each gene was calculated based on the length of the gene and reads count mapped to this gene (Bray el at., 2014).

**Data availability statement**

The sequence of ITS gene in *P. lilacinum* FDZ8Y1 was submitted to NCBI, accession number PP949835.1. The accession numbers of the ITS sequences used for phylogenetic analysis were OK510227.1 (*P. lilacinum* B1), MH856891.1 (*P. lilacinum* CBS:346.51), MZ359561.1 (*P. sodanum* JMRC: NRZ: 0148), NR_166039.1 (*P. lavendulum* CBS 128677), JF896088.1 (*P. takamizusanensis* DTO 78H9), and AJ786558.1 (*P. atypicola* NHJ2). Genome of the strain FDZ8Y1 have been submitted to the GenBank database under accession number JBETMV000000000.1. Raw sequencing reads for the transcript information in this work were deposited to the NCBI Sequence Read Archive under sequential accession numbers from SRR29499926 to SRR29499934. All data are publicly available.

## Supplementary Figure S1

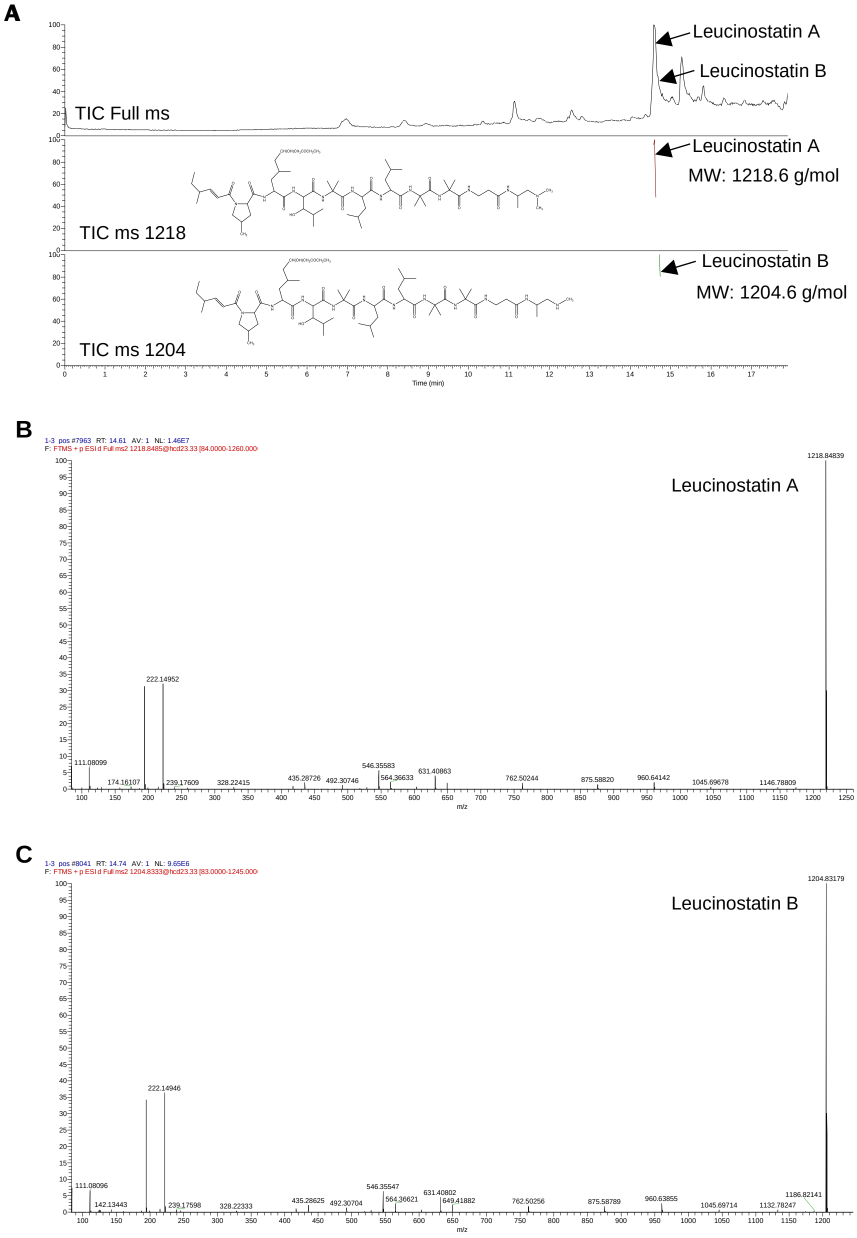


**Supplementary Figure S1.** UPLC-MS/MS diagram of secondary metabolites of *P. lilacinum* FDZ8Y1. (A) Chromatogram by UPLC-MS/MS of *P. lilacinum* FDZ8Y1. The first one was the total ion chromatogram (TIC) of all mass. The second and third one was chromatogram of Leucinostatin A and Leucinostatin B, respectively. (B) MS/MS spectrum of Leucinostatin A (*m/z* 1218). (C) MS/MS spectrum of Leucinostatin

B (*m/z* 1204).

**Supplementary Figure S2**

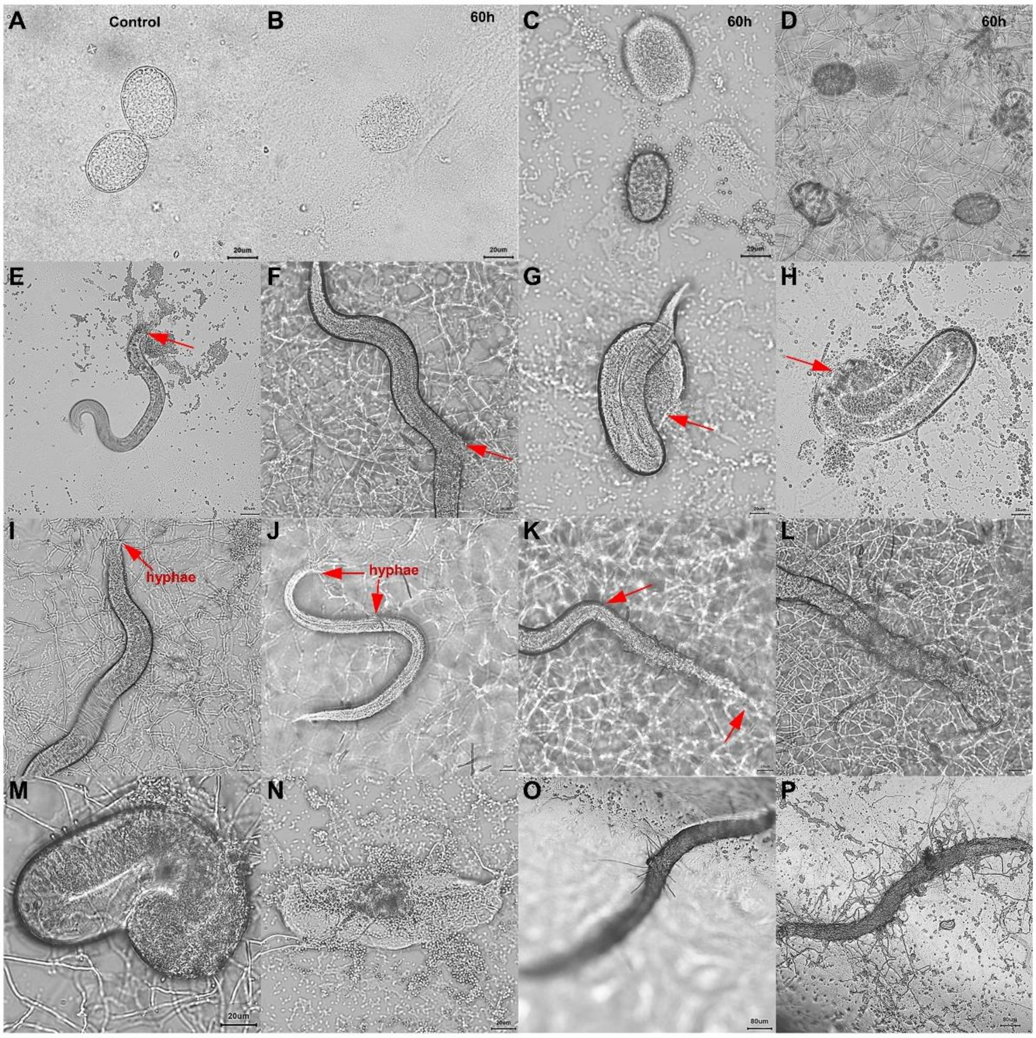


**Supplementary Figure S2.** Nematicidal activity of hadal-derived fungus *P. lilacinum* FDZ8Y1. (A-D) Nematicidal activity of *P. lilacinum* FDZ8Y1 on *C. elegans* eggs. The egg in (A) was a negative control without treatment, while eggs in (B-D) were treated with *P. lilacinum* FDZ8Y1. (E-P) Nematicidal activity of *P. lilacinum* FDZ8Y1 on *C. elegans*. The spores infect the nematode by adhering to the host's cuticle, where they germinate and begin to form hyphae that wrap around and envelop the nematode, eventually suffocating it, penetrating and digesting it. The spores adhered to cuticle and hyphae wrap around the nematode were marked with red arrows.

**Supplementary Figure S3**

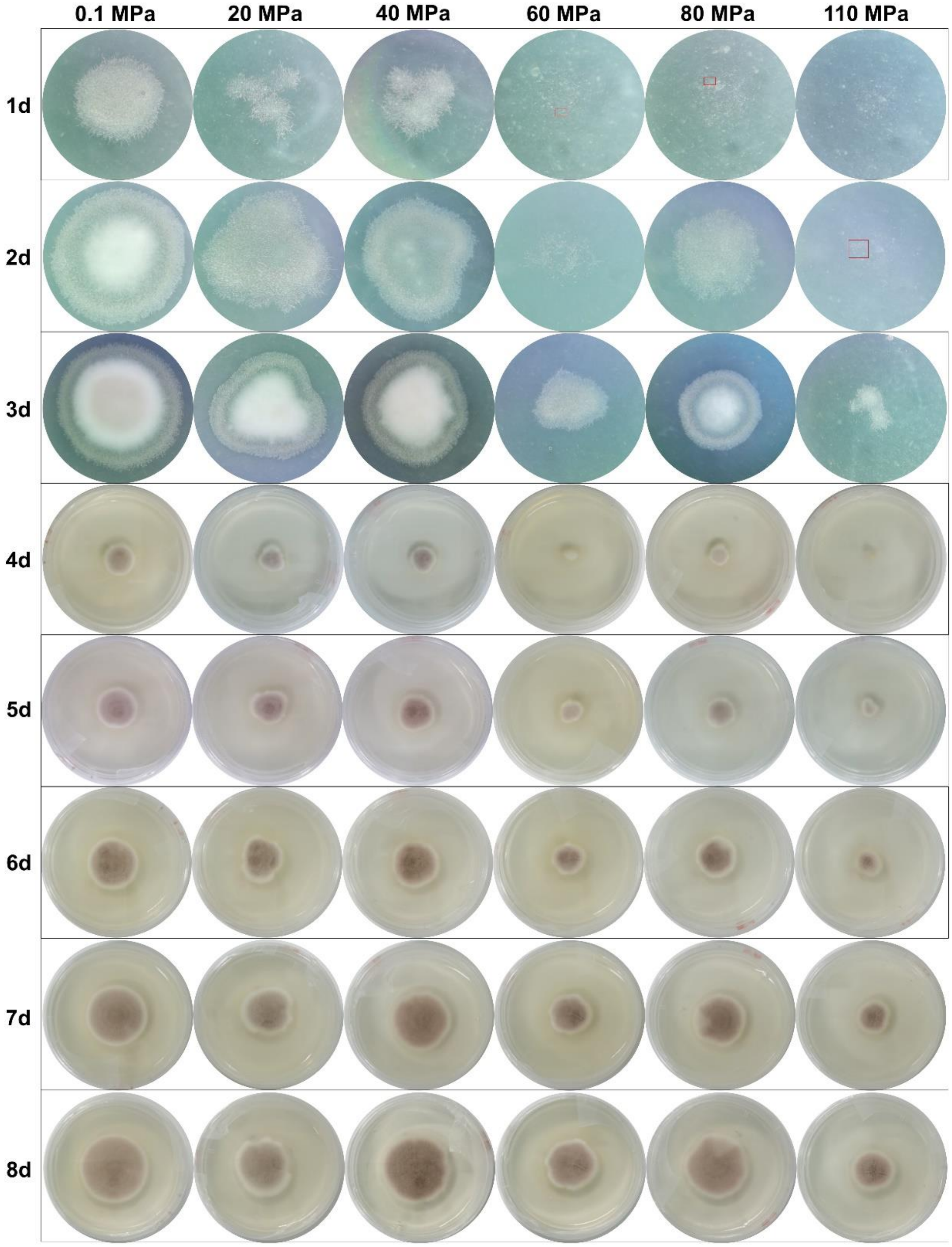


**Supplementary Figure S3.** The colony morphology of *P. lilacinum* FDZ8Y1 spores cultured on PDA after treated with elevated HHP. The single colonies cultured on PDA for 1-3 days were observed using a stereomicroscope (Olympus Corporation, Japan).

**Supplementary Figure S4**

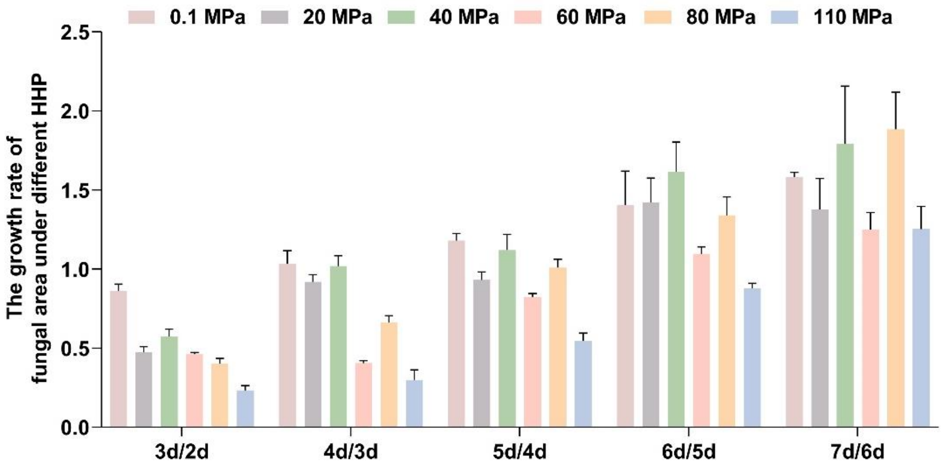


**Supplementary Figure S4.** The growth rate of *P. lilacinum* FDZ8Y1 cultured on PDA after treating with different pressure. The histogram was plotted in GraphPad Prism 8.

**Supplementary Figure S5**

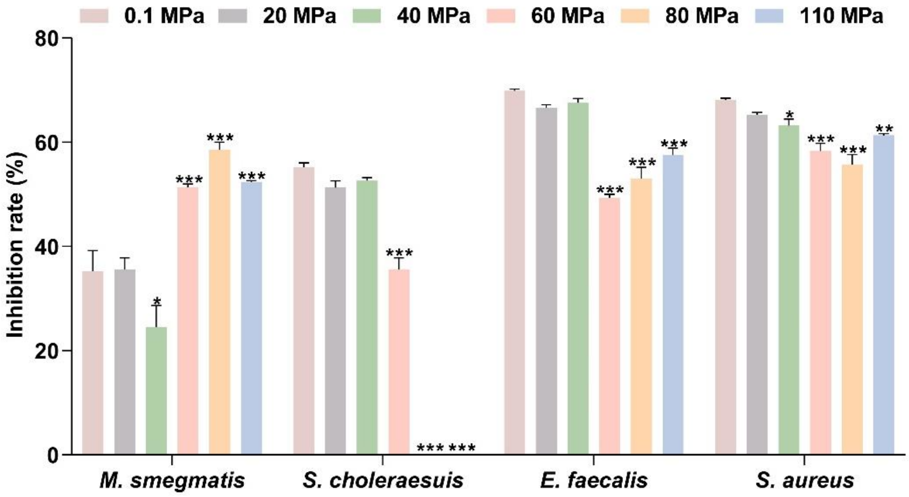


**Supplementary Figure S5.** Inhibitory rates of secondary metabolites produced by *P. lilacinum* FDZ8Y1 after treated with elevated HHP. Statistical significance of the treatment groups (20-110 MPa) compared with the control group (0.1 MPa). The histogram was plotted in GraphPad Prism 8.

**Supplementary Figure S6**

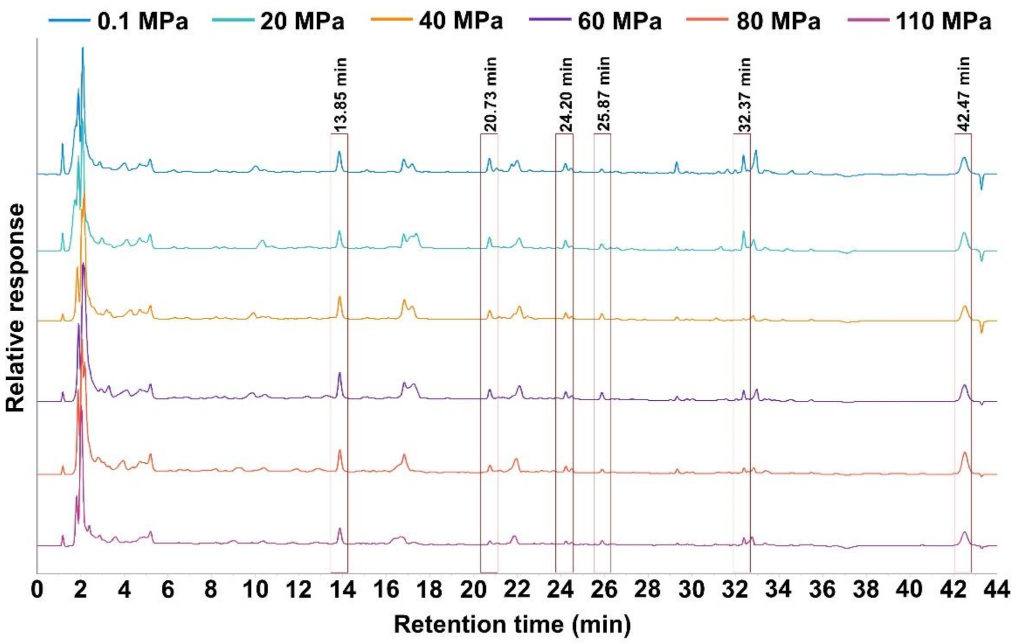


**Supplementary Figure S6.** HPLC analysis of secondary metabolites produced by *P. lilacinum* FDZ8Y1 after treated with elevated HHP. The part highlighted by the red box represents the selected retention time.

**Supplementary Figure S7**

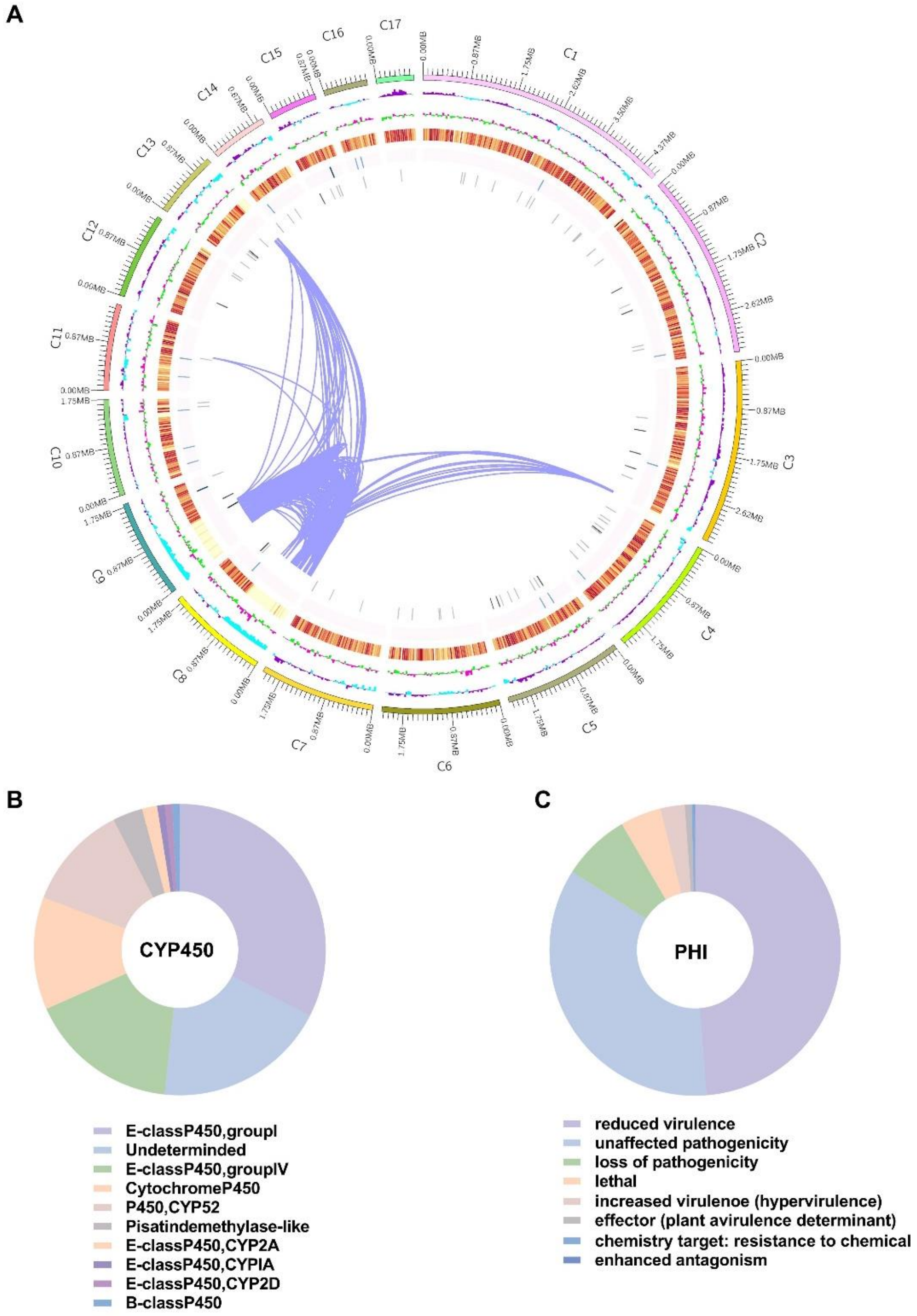


**Supplementary Figure S7.** General structure and functional genes classification of *P. lilacinum* FDZ8Y1. (A) Circular whole genome diagram of *P. lilacinum* FDZ8Y1. The outermost layer is the position coordinates. From the outer circle to the inner circle is GC content (purple: > mean value, blue: < mean value). GC skew (the specific algorithm= (G-C) / (G+C); pink: > 1, green: < 1). Gene density (four circles were taken

inward from orange, representing the numerical value of coding genes, rRNA, snRNA, and tRNA, respectively). Gene duplication (regions with similarity greater than 90% of 8 kb sequences were connected by purple lines). The diagram was drawn using Circos software. (B) Composition and relative abundance of cytochrome P450 (CYP450) genes of *P. lilacinum* FDZ8Y1. (C) Composition and relative abundance of pathogen host interactions (PHI) genes of *P. lilacinum* FDZ8Y1. The pie chart was drawn using GraphPad Prism 8.

**Supplementary Figure S8**

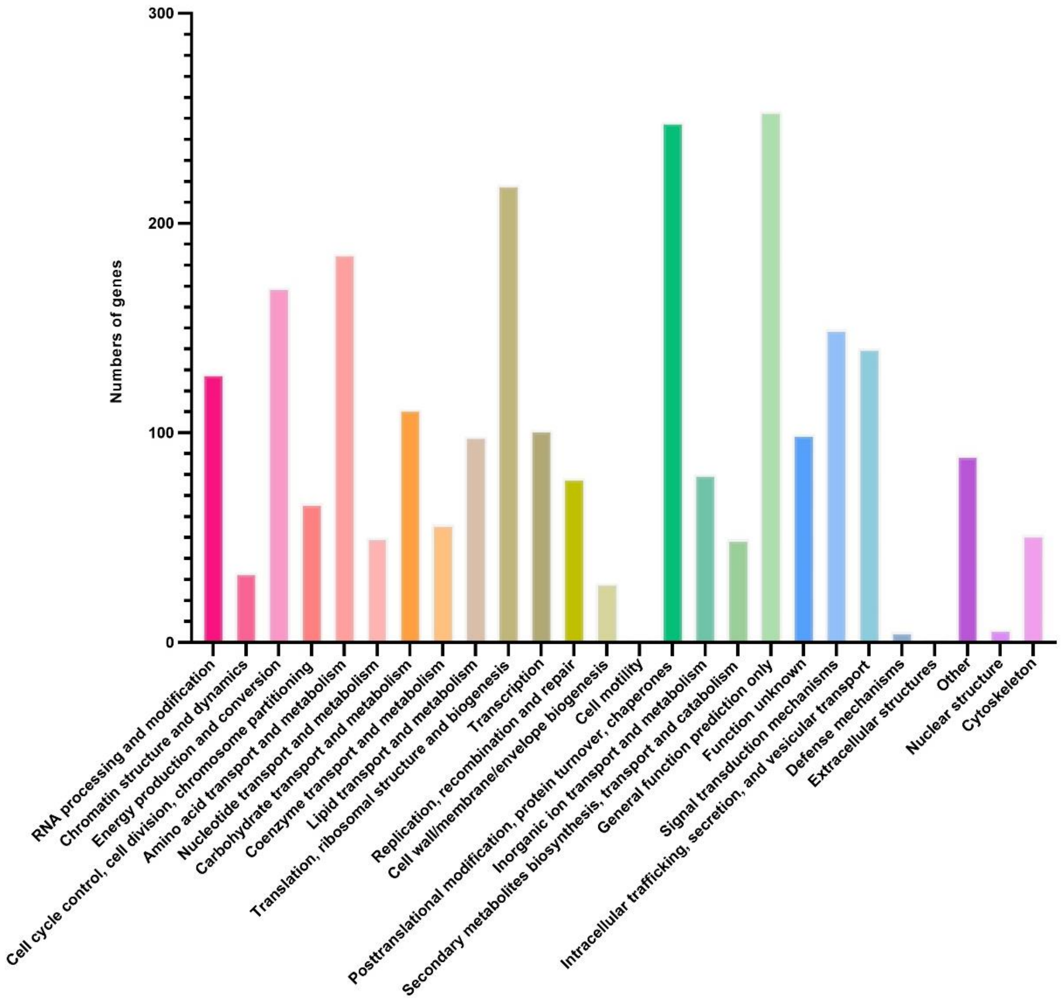


**Supplementary Figure S8.** The Eukaryotic Orthologous Groups of protein (KOG) function annotation of *P. lilacinum* FDZ8Y1. A total of 2492 protein-coding genes were predicted to display KOG functional annotation. Except for the category “General function prediction only”, the majority of the predicted genes were related to the following categories: “Posttranslational modification, protein turnover, chaperones”, “Translation, ribosomal structure and biogenesis”, “Amino acid transport and metabolism”, and “Energy production and conversion”. The bar chart was drawn using GraphPad Prism 8.

**Supplementary Figure S9**

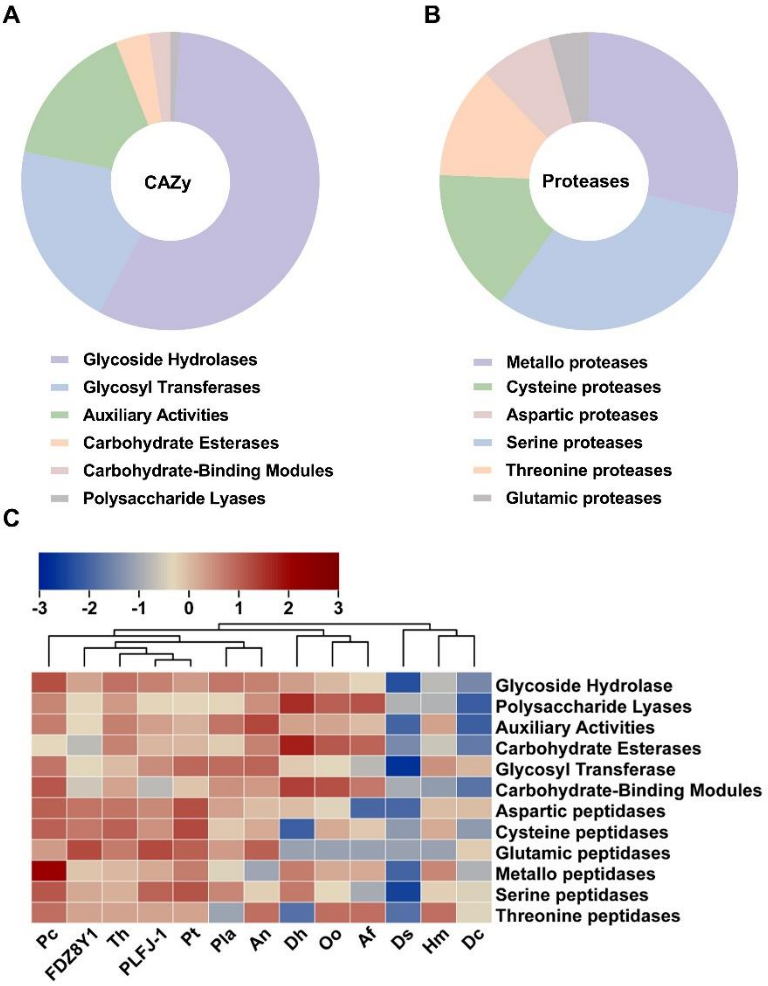


**Supplementary Figure S9.** Functional genes classification and comparison of CAZymes and peptidases families in *P. lilacinum* FDZ8Y1. (A) Composition and relative abundance of CAZyme genes of *P. lilacinum* FDZ8Y1. Glycoside hydrolases (GH) (219), followed by glycosyl transferases (GT, 78) and auxiliary activity proteins (AA, 61), polysaccharide lyases (PL, 4), carbohydrate-binding modules (CBM, 9) and carbohydrate esterases (CE, 14). (B) Composition and relative abundance of peptidase genes of *P. lilacinum* FDZ8Y1. Serine peptidase (36 genes), metallo protease (33 genes) and cysteine protease (18 genes). (C)Hierarchical clustering of CAZYmes and peptidases families in 13 fungi. FDZ8Y1/PLFJ-1, *P. lilacinum*; Pt, *P. takamizusanense*; Pla, *P. lavendulum*; Pc, P. *chlamydosporia*; An, *A. niger*; Hm, *H. minnesotensis*; Th, *T.*

*harzianum*; Dc, *D. coniospora*; Ds, *D. stenobrocha*; Oo, *O. oligospora*; Dh, *D. haptotyla*; Af, *A. flagrans*. The pie chart was drawn using GraphPad Prism 8. The heatmap was drawn using TBtools software.

**Supplementary Figure S10**

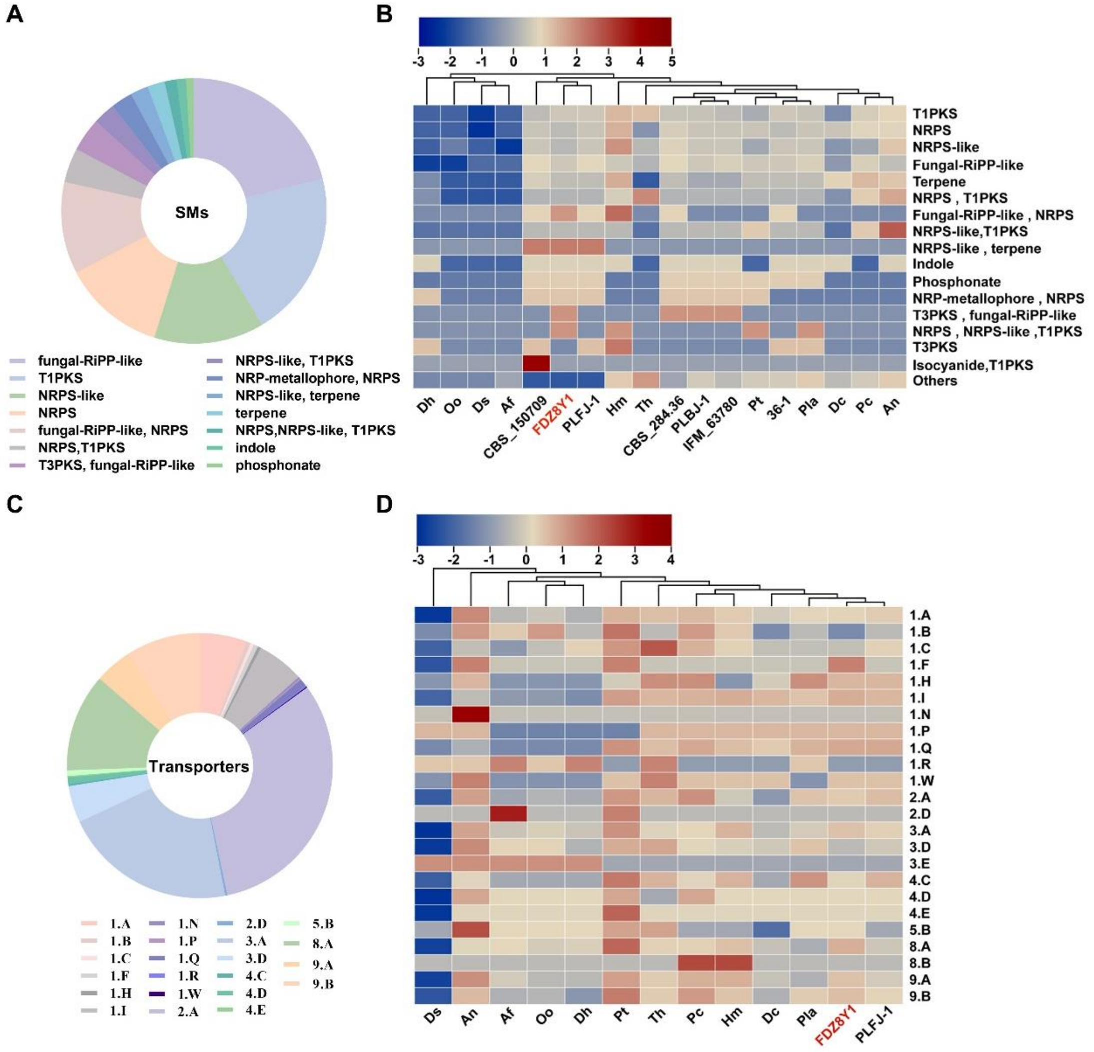


**Supplementary Figure S10.** Functional genes classification and comparison of secondary metabolite biosynthesis gene clusters and peptidases families in *P. lilacinum* FDZ8Y1. (A) Composition and relative abundance of secondary metabolite biosynthesis gene clusters of *P. lilacinum* FDZ8Y1. (B) Hierarchical clustering of secondary metabolite biosynthesis gene clusters in 18 fungi. (C) Composition and relative abundance of transporter genes of *P. lilacinum* FDZ8Y1. 735 transporters in the *P. lilacinum* FDZ8Y1 genome were identified. Porters (uniporters, symporters, antiporters) (31.7%) and P-P-bond-hydrolysis-driven transporters (21.1%) were the most abundant transporters. (D) Hierarchical clustering of transporter genes in 13 fungi. The content of transporters was similar to that of egg-parasitic fungi and cyst-parasitic

fungi, but much higher than that of nematode-trapping fungi. FDZ8Y1 / PLFJ-1 / PLBJ-1 / CBS_150709 / CBS_284.36 / IFM_63780 / 36-1, *P. lilacinum*; Pt, *P. takamizusanense*; Pla, *P. lavendulum*; Pc, P. *chlamydosporia*; An, *A. niger*; Hm, *H. minnesotensis*; Th, *T. harzianum*; Dc, *D. coniospora*; Ds, *D. stenobrocha*; Oo, *O. oligospora*; Dh, *D. haptotyla*; Af, *A. flagrans*. 1.A, α-Type Channels; 1.B, β-Barrel Porins; 1.C, Pore-Forming Toxins (Proteins and Peptides); 1.F, Vesicle Fusion Pores; 1.H, Paracellular Channels; 1.I, Membrane-bounded Channels; 1.N, Cell Fusion Pores; 1.P, Non-Envelop Virus Penitration Complex; 1.Q, Fungal Septal Pores; 1.R, Membrane Contact Site (MCS) for Interorganellar Transport; 1.W, Phage Portal Protein Subclass; 2.A, Porters (uniporters, symporters, antiporters); 2.D, Transcompartment Lipid Carrier; 3.A, P-P-bond-hydrolysis-driven transporters; 3.D, Oxidoreduction-driven transporters; 3.E, Light absorption-driven transporters; 4.C, Acyl CoA ligase-coupled transporters; 4.D, Polysaccharide Synthase/Exporters; 4.E, Vacuolar Polyphosphate Polymerase-catalyzed Group Translocators; 5.B, Transmembrane 1-electron transfer carriers; 8.A, Auxiliary transport proteins; 8.B, Ribosomally synthesized protein/peptide toxins/agonists that target channels and carriers; 9.A, Recognized transporters of unknown biochemical mechanism; 9.B, Putative transport proteins. The pie chart was drawn using GraphPad Prism 8. The heatmap was drawn using TBtools software.

**Supplementary Figure S11**

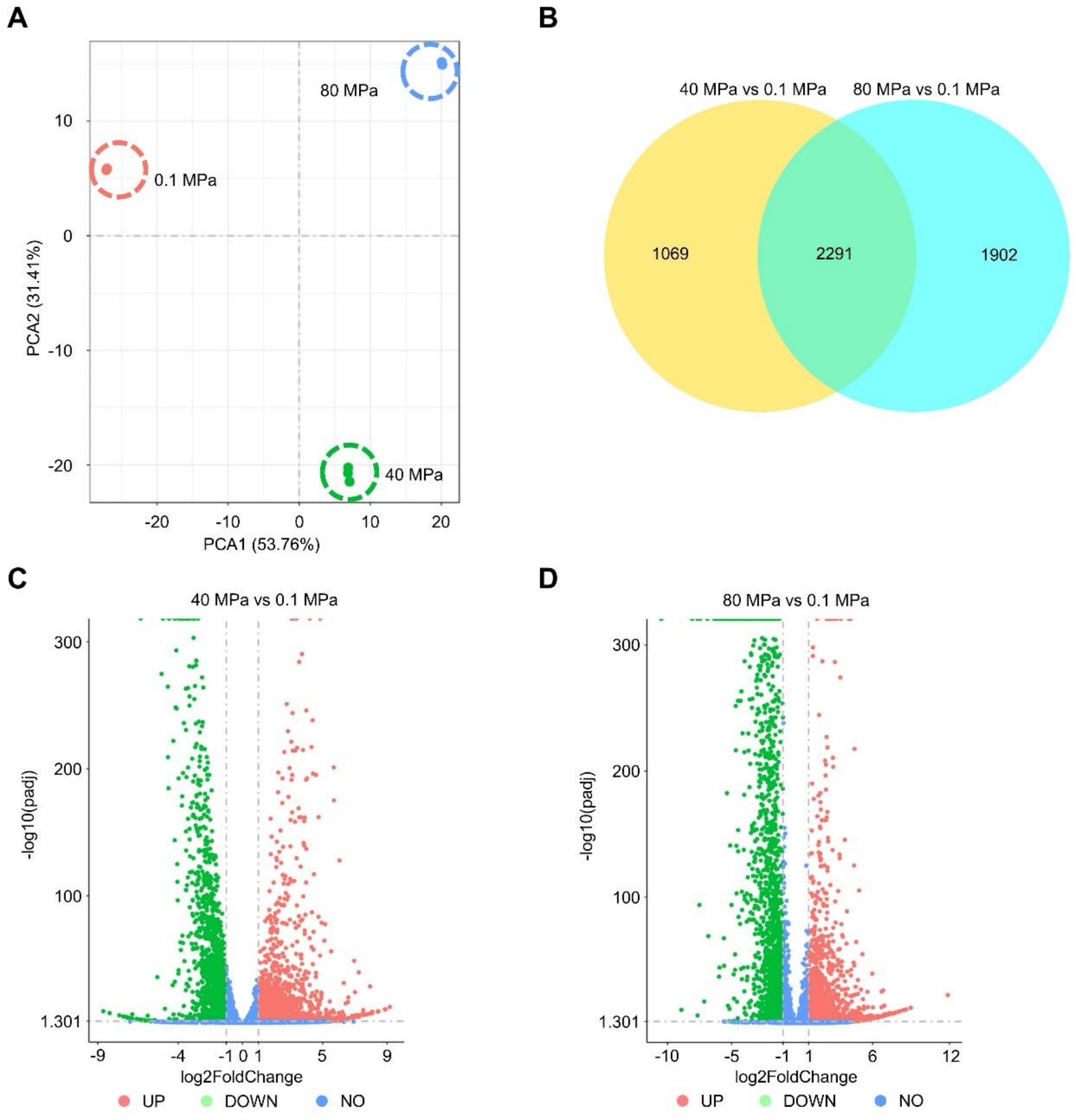


**Supplementary Figure S11.** Transcriptome overview. (A) principal component analysis (PCA) of the gene expression profiles in different groups. PCA was drawn in Rstudio using ggplot2 package (Version 3.0.3). (B-D) Numbers of differential expression genes ($\log_2$ foldchange > 1 or < -1) showed in venn diagram and volcano plot. A total of 3,360 DEGs were detected in *P. lilacinum* FDZ8Y1 cultured under 40 MPa, including 1,758 up-regulated genes and 1,602 down-regulated genes, while more

DEGs in the group of 80 MPa (4,193 DEGs with 2,154 up-regulated and 2,039 down-regulated genes). A total of 2,291 DEGs were shared in two groups. 0.1 MPa represents *P. lilacinum* FDZ8Y1 cultured under 0.1 MPa; 40 MPa represents *P. lilacinum* FDZ8Y1 cultured under 40 MPa; 80 MPa represents *P. lilacinum* FDZ8Y1 cultured under 80 MPa. Data was analyzed in E Venn (http://www.ehbio.com/test/venn/#/). Volcano plot was drawn in NoveMagic (https://magic.novogene.com/customer/main#/omicslist/7546fdc89e6d0d5c4e11b83aee85ee8f). The pie chart was drawn using GraphPad Prism 8. The heatmap was drawn using TBtools software.

## Supplementary Figure S12

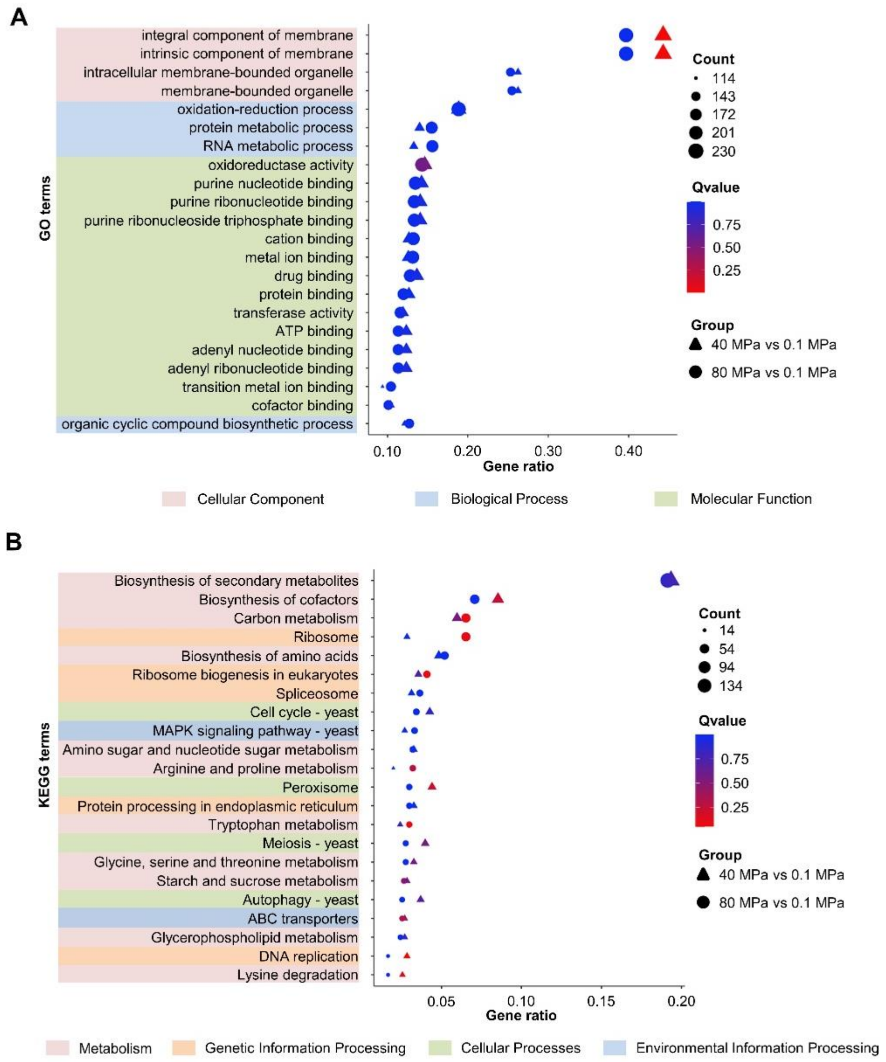


**Supplementary Figure S12.** Enrichment analysis of DEGs in two groups. Top 20 terms in (A) GO and (B) KEGG enriched from DEGs under 40 MPa and 80 MPa. Figure was drawn by ImageGP (https://www.bic.ac.cn/BIC/).

## Supplementary Figure S13

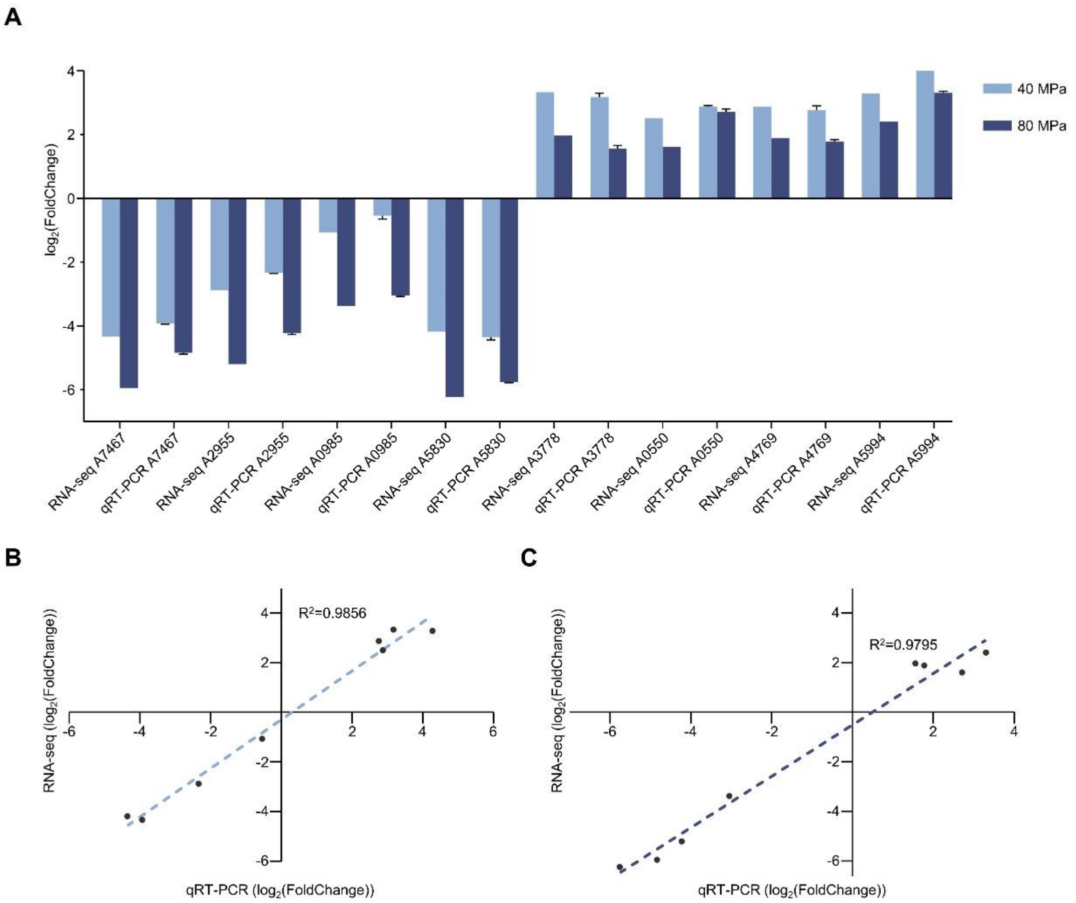


**Supplementary Figure S13.** Relative expression levels of differently expressed genes validated by qRT-PCR. (A) 8 genes were selected to verify the results of RNA-seq by qRT-PCR.

qRT-PCR was performed using 7500 Real-Time PCR System (Applied Biosystems, US). (B-C) The value correlations between RNA-seq and qRT-PCR in (B) 40 MPa and (C) 80 MPa group. The correlations between RNA-seq and qRT-PCR exhibited well ($R^2 > 0.95$). The picture was plotted in GraphPad Prism 8.

## References


Ashburner, M., Ball, C.A., Blake, J.A., Botstein, D., Butler, H., Cherry, J.M., et al. (2000). Gene ontology: tool for the unification of biology. The Gene Ontology Consortium. *Nat. Genet. 25*, 25-29. doi: 10.1038/75556.

Benson, G. (1999). Tandem repeats finder: a program to analyze DNA sequences. *Nucleic Acids Res. 2*, 573-580. doi: 10.1093/nar/27.2.573.

Blin, K., Shaw, S., Augustijn, H.E., Reitz, Z.L., Biermann, F., Alanjary, M., et al. (2023). antiSMASH 7.0: new and improved predictions for detection, regulation, chemical structures and visualisation. *Nucleic Acids Res. 51*, W46-W50. doi: 10.1093/nar/gkad344.

Bray, N.L., Pimentel, H., Melsted, P., and Pachter, L.S. (2015). Near-optimal RNA-Seq quantification. *ArXiv* abs/1505.02710. doi: 10.48550/arXiv.1505.02710.

Buchfink, B., Reuter, K., and Drost, H.G., (2021). Sensitive protein alignments at tree-of-life scale using DIAMOND. *Nat. Methods 18*, 366-368. doi: 10.1038/s41592-021-01101-x.

Finn, R.D., Bateman, A., Clements, J., Coggill, P., Eberhardt, R.Y., Eddy, S.R., et al. (2014). Pfam: the protein families database. *Nucleic Acids Res. 42*, D222-D230. doi: 10.1093/nar/gkt1223.

Kanehisa, M., Araki, M., Goto, S., Hattori, M., Hirakawa, M., Itoh, M., et al. (2008). KEGG for linking genomes to life and the environment. *Nucleic Acids Res. 36*, D480-D484. https://doi.org/10.1093/nar/gkm882.

Kohler, A., Kuo, A., Nagy, L.G., Morin, E., Barry, K.W., Buscot, F., et al. (2015). Convergent losses of decay mechanisms and rapid turnover of symbiosis genes in *mycorrhizal mutualists*. *Nat. Genet. 47*, 410-415. https://doi.org/10.1038/ng.3223.

Koonin, E.V., Fedorova, N.D., Jackson, J.D., Jacobs, A.R., Krylov, D.M., Makarova, K.S., et al. (2004). A comprehensive evolutionary classification of proteins encoded in complete eukaryotic genomes. *Genome Biol. 5*, R7.

https://doi.org/10.1186/gb-2004-5-2-r7.

Lagesen, K., Hallin, P., Rødland, E.A., Staerfeldt, H.H., Rognes, T., and Ussery, D.W. (2007). RNAmmer: consistent and rapid annotation of ribosomal RNA genes. *Nucleic Acids Res. 35*, 3100-3108. doi: 10.1093/nar/gkm160.

Li, W., Jaroszewski, L., and Godzik, A. (2002). Tolerating some redundancy significantly speeds up clustering of large protein databases. *Bioinformatics 18*, 77-82. doi: 10.1093/bioinformatics/18.1.77.

Liao, Y., Smyth, G.K., Shi, W. (2014). featureCounts: an efficient general purpose program for assigning sequence reads to genomic features. *Bioinformatics* (Oxford, England), 30, 923–930. doi: 10.1093/bioinformatics/btt656.

Lowe, T.M., and Eddy, S.R. (1997). tRNAscan-SE: a program for improved detection of transfer RNA genes in genomic sequence. *Nucleic Acids Res. 25*, 955-964. doi: 10.1093/nar/25.5.955.

Mortazavi, A., Williams, B. A., McCue, K., Schaeffer, L., & Wold, B. (2008). Mapping and quantifying mammalian transcriptomes by RNA-Seq. *Nat. Methods* 5, 621–628. doi: 10.1038/nmeth.1226.

Rawlings, N.D., O'Brien, E., and Barrett, A.J. (2002). MEROPS: the protease database. *Nucleic Acids Res. 30*, 343-346. doi: 10.1093/nar/30.1.343.

Reiner, J., Pisani, L., Qiao, W., Singh, R., Yang, Y., Shi, L., et al. (2018). Cytogenomic identification and long-read single molecule real-time (SMRT) sequencing of a *Bardet-Biedl Syndrome 9* (*BBS9*) deletion. *npj Genomic Med. 3*, 3. doi: 10.1038/s41525-017-0042-3.

Saha, S., Bridges, S., Magbanua, Z.V., and Peterson, D.G., (2008). Empirical comparison of ab initio repeat finding programs. *Nucleic Acids Res. 36*, 2284-2294. doi: 10.1093/nar/gkn064.

Saier, M.H., Reddy, V.S., Moreno-Hagelsieb, G., Hendargo, K.J., Zhang, Y., Iddamsetty, V., et al. (2021). The Transporter Classification Database (TCDB): 2021 update. *Nucleic Acids Res. 49*, D461-D467. doi: 10.1093/nar/gkaa1004.

Stanke, M., Diekhans, M., Baertsch, R., and Haussler, D. (2008). Using native and syntenically mapped cDNA alignments to improve *de novo* gene finding. *Bioinformatics 24*, 637-644. doi: 10.1093/bioinformatics/btn013.

Walker, B.J., Abeel, T., Shea, T., Priest, M., Abouelliel, A., Sakthikumar, S., et al. (2014). Pilon: an integrated tool for comprehensive microbial variant detection and genome assembly improvement. *PLoS One 9*, e112963. doi: 10.1371/journal.pone.0112963.

Wick, R.R., Judd, L.M., Gorrie, C.L., and Holt, K.E., (2017). Unicycler: Resolving bacterial genome assemblies from short and long sequencing reads. *PLoS Comput*. Biol. *13*, e1005595. doi: 10.1371/journal.pcbi.1005595.

Zheng, J., Ge, Q., Yan, Y., Zhang, X., Huang, L., and Yin, Y., (2023). dbCAN3: automated carbohydrate-active enzyme and substrate annotation. N*ucleic Acids Res. 51*, W115-W121. doi: 10.1093/nar/gkad328.
